# Neuronopathic Gaucher disease models reveal defects in cell growth promoted by Hippo pathway activation

**DOI:** 10.1038/s42003-023-04813-2

**Published:** 2023-04-19

**Authors:** Daria Messelodi, Silvia Strocchi, Salvatore Nicola Bertuccio, Pascale Baden, Valentina Indio, Federico M. Giorgi, Alberto Taddia, Salvatore Serravalle, Sabrina Valente, Alessio di Fonzo, Emanuele Frattini, Roberto Bernardoni, Annalisa Pession, Daniela Grifoni, Michela Deleidi, Annalisa Astolfi, Andrea Pession

**Affiliations:** 1 Department of Medical and Surgical Sciences, University of Bologna, 40138 Bologna, Italy; 2 Laboratory of Translational Research, USL-IRCCS of Reggio Emilia, 42123 Reggio Emilia, Italy; 3 German Center for Neurodegenerative Diseases (DZNE), Tübingen, 72076 Germany; 4 Hertie Institut for Clinical Brain Research, University of Tübingen, 72076 Tübingen, Germany; 5 Department of Veterinary Medical Sciences, University of Bologna, 40064 Ozzano dell’Emilia (BO), Italy; 6 Department of Pharmacy and Biotechnology, University of Bologna, 40126 Bologna, Italy; 7 Pediatric Unit, IRCCS Azienda Ospedaliero-Universitaria di Bologna, 40138 Bologna, Italy; 8 Neurology Unit, Fondazione IRCCS Ca’ Granda Ospedale Maggiore Policlinico, 20122 Milan, Italy; 9 Alma Mater University of Bologna, 40126 Bologna, Italy; 10 Department of Life, Health and Environmental Sciences (MeSVA), University of L’Aquila, 67100 L’Aquila, Italy; 11 Institut Imagine, INSERM UMR1163, 75015 Paris, France

**Keywords:** Mechanisms of disease, Lipid-storage diseases, Metabolic disorders

## Abstract

Gaucher Disease (GD), the most common lysosomal disorder, arises from mutations in the *GBA1* gene and is characterized by a wide spectrum of phenotypes, ranging from mild hematological and visceral involvement to severe neurological disease. Neuronopathic patients display dramatic neuronal loss and increased neuroinflammation, whose molecular basis are still unclear. Using a combination of *Drosophila dGBA1b loss-of-function* models and GD patient-derived iPSCs differentiated towards neuronal precursors and mature neurons we showed that different GD- tissues and neuronal cells display an impairment of growth mechanisms with an increased cell death and reduced proliferation. These phenotypes are coupled with the downregulation of several Hippo transcriptional targets, mainly involved in cells and tissue growth, and YAP exclusion from nuclei. Interestingly, Hippo knock-down in the GBA-KO flies rescues the proliferative defect, suggesting that targeting the Hippo pathway can be a promising therapeutic approach to neuronopathic GD.

## Abstract (web summary)

A combination of *Drosophila dGBA1b* loss-of-function models and Gaucher Disease (GD) patient-derived iPSCs reveals an impairment in GD neuronal cell growth and that Hippo pathway hyperactivation contributes to the impairment.

## Introduction

Gaucher disease (GD) is the most common lysosomal storage disorder, occurring 1 in 40,000–60,000 births^1^. The causative genetic alterations involve the *GBA1* gene, encoding the lysosomal enzyme ß-glucocerebrosidase (GCase). More than 400 *GBA1* mutations have already been described, leading to an altered enzymatic activity and, in most cases, to systemic manifestations that involve the spleen, liver, bone marrow, lungs, and central nervous system^2–4^. GD is clinically classified into three major groups based on the absence (GD type 1) or presence and rate of progression of neurological manifestations (GD type 2 and 3)^5–9^. However, these categories are not strictly separated and, especially among the neuronopathic forms, there are many cases presenting an intermediate phenotype. The wide range of symptoms, spanning from very mild hematological and visceral phenotypes to severe neurological involvement, and the absence of a clear phenotype-genotype correlation supports the idea that other players interact with *GBA1* mutations to give rise to the clinical picture^10–12^. To further complicate the situation, some evidences prove the role of *GBA1* mutations as a major genetic risk factor for the development of Parkinson’s disease^13^.

Neuronopathic GD (nGD) is a relatively rare condition among all the patients affected by the disease, but it is certainly the most severe and limiting form. nGD is characterized by dramatic neuronal loss, microgliosis, and neuroinflammation, with the activation of type I interferon response^14,15^. *GBA1* mutations were also observed to cause endoplasmic reticulum (ER) stress and autophagic defects in the neural compartment^16,17^.

Different studies conducted in mammals and flies shed light on the defective communication between the two usually coupled mechanisms of ER stress and unfolded protein response (UPR). The upregulation of these pathways, combined with vesicular trafficking impairment, lead to alterations in dopaminergic neurons and developmental defects^18,19^. Nevertheless, the biological mechanisms leading to central nervous system involvement are still unclear, and no effective therapeutic options are available.

In this study, we exploited an integrated model approach based on *Drosophila melanogaster* and GD patient-derived induced pluripotent stem cells (iPSCs) to investigate the cellular and molecular basis of this condition, analysing the altered activation of signaling pathways involved in neurodegeneration-related mechanisms.

Human *GBA1* and the *Drosophila* homolog, *dGBA1b*, share high structural and functional identity^20^, and most GD pathological traits are reproduced in the fly model^19,21–23^. Conversely, iPSC-derived neuronal and macrophagic cells are valuable tools to study specific cell populations in GD, since they efficiently recapitulate disease characteristics and biochemical features^24–27^.

Through gene expression profile analysis, we show that genes related to cell death and proliferation are transcriptionally impaired in GBA-KO fly brains, proving that cell growth/death balance is altered, thus leading to a decrease in cell proliferation and an increase in cell death in both *Drosophila* tissue and GD iPSC-derived neuronal cells. Therefore, we hypothesize that the alteration of growth/death balance contributes to the nGD outcome.

One of the master regulators of these processes is the Hippo pathway, deeply studied in association with cancer onset, but also recently connected to neurodegenerative syndromes^28–37^. The Hippo signaling has been established in neuroinflammation, neuronal cell differentiation, and neuronal death, where its role in mechano-transduction and regulation of cell growth are catching more and more attention^38^. In microglia cells, the lower expression of YAP induced a proinflammatory status that increased the Aβ1-42 complex and tau phosphorylation in Alzheimer patients^39^. Moreover, it has been demonstrated that hyper-Hippo-related necrosis is peculiar to the initial stage of Alzheimer’s disease^40^. The hyper-activation of upstream Hippo pathway components has been found to cause the inactivation of the downstream effector Yorkie (Yki) in flies, YAP in humans, and the block of growth signals. This resulted in neuroinflammation and neuronal cell death both in *Drosophila* and in mammalian systems, associated with different pathologic background such as astrogliosis and Huntington’s disease^41–45^.

This study expands the knowledge on nGD, demonstrating the imbalance of growth/death physiological mechanisms. In the GBA-KO condition, the strong downregulation of YAP/Yki targets and the restoration of cell proliferation following knockdown (KD) of the Hippo kinase component, suggest that Hippo pathway hyper-activation can play a role in nGD neuronal loss and death.

## Results

### *dGBA1b* deficiency causes neurodegenerative phenotypes in *Drosophila*

**Fig. 1:**
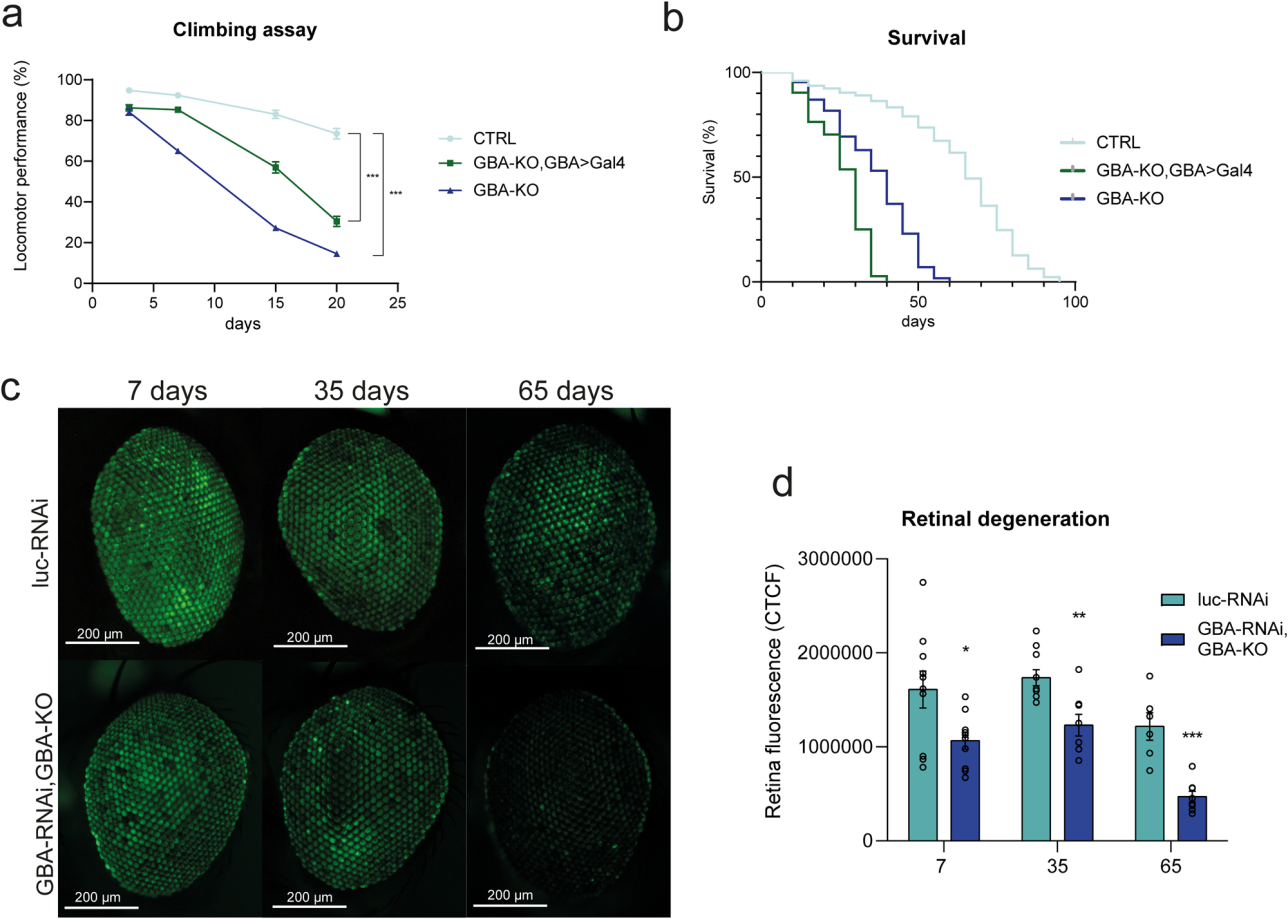
GCase deficiency causes retinal degeneration and behavioral deficits associated to neurodegeneration. **a** GBA-LoF models (GBA-KO and GBA-KO, GBA > Gal4) show a significant impairment of climbing abilities. Climbing assay was performed on 150 flies at 3, 7, 15, and 20 days. w^1118^ are used as control flies (CTRL). Two ways ANOVA test and unpaired *t*-test comparing CTRL and GBA-KO at the four time points resulted in statistical significance (***p < 0.001). **b** GBA-LoF models show heavily reduced lifespan. Overall survival was calculated with the Kaplan–Meyer curve on 150 flies for each genotype. Log rank *p* value <0.001. **c** Representative Z-stack acquisition of adult eyes expressing *luc*-RNAi, used as control, or GBA-RNAi, GBA-KO under the retina-specific promoter *GMR*. Time points: 7, 35, and 65 days from hatching. Scale bar: 200 µm **d** Fluorescence quantification of adult eyes expressing *luc*-RNAi or GBA-RNAi under the *GMR* promoter using the corrected total cell fluorescence (CTCF) method. *n* = 3 independent measurements of three flies each. The graph shows mean values and SEM. *p < 0.05; **p < 0.01; ***p < 0.001.

In order to investigate reliable and informative model systems of nGD, different *Drosophila* GD models have been exploited. We took advantage of two GBA1 loss-of-function (LoF) models. The first one is the homozygous *GBA*-KO model, generated by the Partridge group^22^, in which both *dGBA1b* alleles were deleted through the introduction of a premature stop codon within the coding sequence. The second one is the heterozygous GBA-KO, GBA > Gal4 model, where *dGBA1b* was inactivated by the insertion of a Gal4 construct under the control of the endogenous promoter. Both models cause a severe LoF phenotype^46^. Although flies do not show morphological alterations and are viable and fertile, the evaluation of the climbing ability, commonly used to estimate the cognitive deficit, revealed significant impairment of climbing skills (Fig. 1a). In addition, lifespan was significantly reduced in both LoF models compared to the CTRL flies (w^1118^), with a median overall survival of 40 and 30 days for GBA-KO and GBA-KO, GBA > Gal4, respectively, with respect to 60 days for CTRL flies (Fig. 1b).

To evaluate the GBA1 LoF-related degeneration of neuronal tissue, we have monitored the flies’ ommatidia status. The heterozygous GBA-RNAi, GBA-KO flies, expressing one copy of UAS-shRNA against the endogenous d*GBA1b* in the adult retina under the *GMR* > Gal4 promoter (or Luciferase as a control), were used. This system was combined with a membrane-targeted green fluorescent protein (mCD8-GFP) to monitor the neurotoxic protein-dependent degeneration of *Drosophila* eyes^47^. We observed a significant decrease of fluorescence in the GBA-RNAi/GBA-KO model with respect to the control reference at each time point during the aging of the flies (Fig. 1c, d). These data confirm the correlation between the d*GBA1b* LoF and the occurrence of a neurodegenerative phenotype in different *Drosophila* GD models.

### GD *Drosophila* tissues display impairment of the growth/death mechanism

**Fig. 2:**
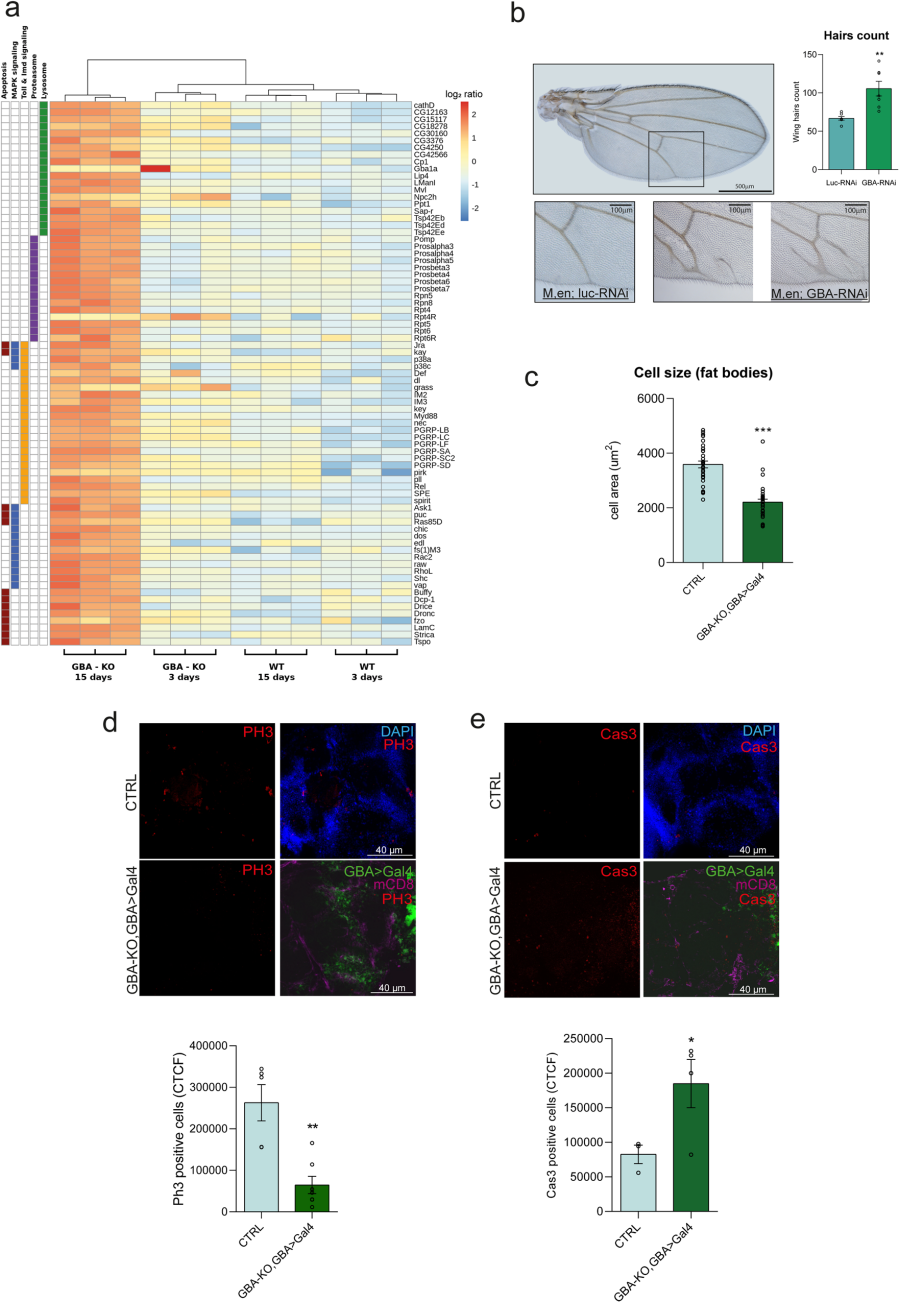
Lack of *dGBA1b* leads to growth/differentiation impairment. **a** Heatmap of the genes belonging to the significantly over-represented pathways in the comparison of CTRL and GBA-KO fly brains 3 and 15 days after hatching. Pathway classification is shown on the left. **b** A wild-type adult wing is displayed; the square indicates the area of interest (in the posterior compartment where *en* > *GBA*-RNAi is expressed). Scale bar: 500 µm. The underlying panels show the normal phenotype of the control flies (*en* > *luc*-RNAi) and two representative pictures of *en* > *GBA*-RNAi adult wings showing the impairment of vein development. Scale bar: 100 µm. Count of the wing hairs of multiple areas of seven individuals (each) representative of the *GBA*-RNAi progeny and the *luc*-RNAi. **p < 0.01. **c** Quantification of fat body cell areas demonstrate growth homeostasis impairment in GBA-KO, GBA > Gal4 flies (*n* = 10 flies per condition, from 3 different experiments). ***p < 0.001. **d**, **e** Immunofluorescence on adult brains for PH3 (**d**) and Cas3 (**e**) performed on w^1118^ (CTRL) and GBA-KO, GBA > Gal4 flies (*n* = 3, from two different experiments). In both panels, pictures show PH3/Cas3 in red. For the CTRL, it was chosen to show only the nuclei, stained with DAPI in blue, while in the GBA-KO images, the endogenous GFP refers to GBA > Gal4 cells only. The staining for mCD8 in magenta was added to better define organ boundaries. Scale bar: 40 µm. Quantification of signals has been performed with the CTCF method. *p < 0.05; **p < 0.01. All the graphs show mean values and SEM and statistical significance is indicated as Student’s *t*-test results.

To investigate the molecular mechanisms driving the GBA-LoF-related neurodegeneration, we performed whole transcriptome sequencing of CTRL and GBA-KO *Drosophila* brains. The comparison between the gene expression profile of flies at 15 days after hatching highlighted the significant upregulation of genes related to the lysosome and proteasome functionality, the immune response, and MAPK and apoptosis signaling pathways (Fig. 2a and Supplementary Table 4).

The link between neurodegeneration and innate immune response activation was already described in *Drosophila*^48^ and the alteration of the lysosomal-autophagic pathways as well as the activation of the UPR are typical features of nGD, identified also in *Drosophila* GD models^21,22,49,50^.

Unexpectedly, the overexpression of pathways strictly related to the control of proliferation, differentiation and programmed cell death was particularly interesting, suggesting that an impaired balance between cell growth and cell death mechanisms can be connected with GBA-LoF and nGD pathophysiology.

To test this hypothesis, we evaluated the effect of GBA-RNAi on adult fly wings, whose morphology mirrors developmental alterations^51–54^. We evaluated the presence of modified phenotypes associated with the LoF of GBA, placing the flies in a *Minute* (*M*) context, characterized by the prolongation of developmental time and, therefore, by a worsened phenotype. As expected, induction of *GBA1b*-RNAi under control of the *engrailed* (*en*) promoter on the third chromosome showed an altered pattern of the posterior veins, compared with the anterior veins (Fig. 2b). The posterior veins resulted ramified at the intersection with the wing border in more than 80% of the experimental flies, compared to *luc*-RNAi control population. Focusing the attention on the wing hairs (where each bristle is associated with one cell), we observed an increased number of hairs per area, revealing a significant decrease in average cell dimensions, indicating a growth impairment caused by GBA knockdown in this tissue (Fig. 2b).

To confirm the growth impairment in d*GBA1b* LoF tissues, we evaluated cell dimensions in the fat body tissues of the GBA-KO, GBA > Gal4 flies, since this is one of the tissues most expressing d*GBA1b*. The measure of the cell areas proved that GBA-KO cells are significantly smaller than control cells (Fig. 2c), confirming the evidence obtained in the GBA-RNAi fly wings, and strengthening the assumption of an altered growth mechanism.

Next, we analysed the proliferative status and the apoptotic cell death in adult brains 15 days after hatching, as assessed by Phospho-histone 3 (PH3) and activated Caspase 3 (Cas3) staining, respectively (Fig. 2d, e).

GBA is mainly expressed in the central brain (Supplementary Fig. 1a), so we focussed our analysis on this brain region. Notably, the mitotic activity was significantly reduced in GBA-KO brains compared to CTRL (Fig. 2d), while Cas3 activation resulted increase in the GBA-KO brains (Fig. 2E). These data support the hypothesis that the GBA-KO context is characterized by an impairment of the physiological growth/death balance also at the brain level.

### GD-neural cells display growth defects and an increased cell death rate

**Fig. 3:**
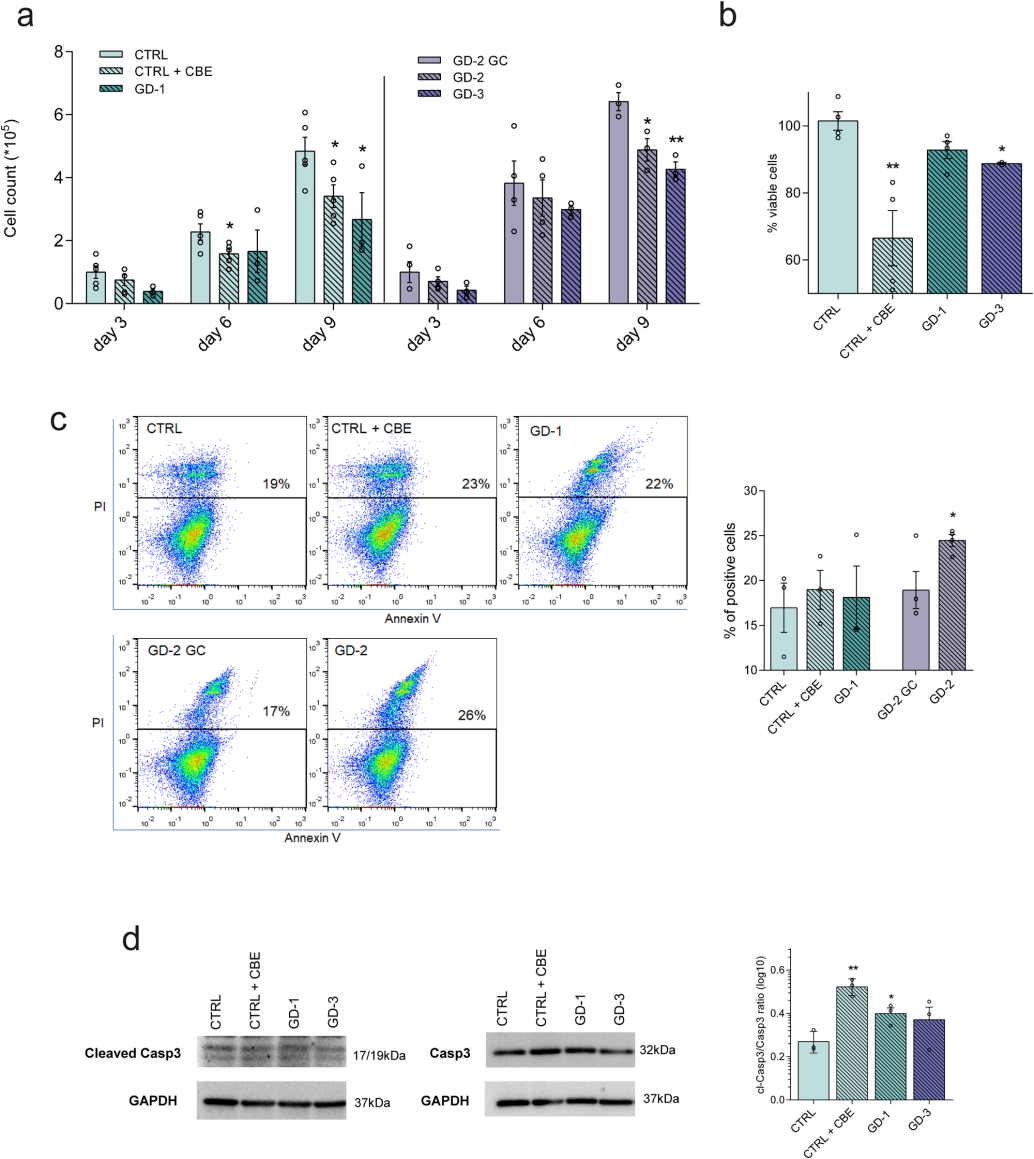
GD NPCs show growth deficit and increased cell death. **a** Evaluation of the cell count of untreated and CBE-treated CTRL and the three GD patients’ NPCs after 3, 6, and 9 days in culture. Cells have been seeded in NPC medium and kept in culture to evaluate the absolute number at the different time points (*n* = 3). CBE-treated and GD-1 NPCs display a significant decrease in cell number after 9 days in culture if compared with CTRL, the same effect is evident also for the two neuronopathic lines compared with the isogenic gene-corrected (GD-2 GC) NPCs. **b** Quantification of absorbance released by metabolic active NPCs after MTT assay, as a direct indicator of viable and proliferating cells. The percentage has been calculated on the media of CTRLs (*n* = 4). **c** Flow cytometry evaluation of NPCs stained with AnnexinV (FITC) and PI after 7 days in culture in standard NPC medium. The quantification of the PI marker positivity suggests a strong effect in cell death induction in the nGD line (GD-2) analysed in this experiment (*n* = 3). **d** Representative Western blot of cleaved Caspase 3 (cleaved Casp3) and total Caspase 3 (Casp3) in GD NPCs (GD-1, GD-3, and CBE treated) and corresponding control (CTRL) with the relative quantification of cleaved Caspase 3/caspase 3 ratio (*n* = 3). All graphs show mean values and SEM, and statistical significance is indicated as *p* value (Student’s *t*-test), *p < 0.05, **p < 0.01.

To evaluate whether the disease-related mechanisms observed in *Drosophila* were also impaired in human neural cells, iPSC-derived neural precursor cells (NPCs) and neurons were employed. Healthy donor (CTRL), GD patient type 1 (GD-1), and GD patient type 3 (GD-3) iPSC lines have been generated, fully characterized (Supplementary Figs. 2, 3) and, in parallel with another GD patient type 3 (GD-2) and the corresponding isogenic gene-corrected line (GD-2 GC), efficiently differentiated. NPCs expressing the characteristic lineage markers (Supplementary Fig. 4a) have been firstly obtained and later differentiated into a population of mature enriched midbrain dopaminergic neurons (Supplementary Fig. 4b). Once differentiated, CTRL iPSC were treated with CBE to induce a GD-like condition. The GCase protein levels and the deficiency of GBA enzymatic activity were assessed (Supplementary Fig. 4c–e). To test the neural cell growth potential, iPSC-derived NPCs were seeded in standard culture conditions and the cell number was evaluated at three different time points (3-6-9 days). All GD lines displayed a significant reduction of the cell count compared to the healthy counterpart (Fig. 3a). This is also observed in measuring cell proliferation and metabolic activity through MTT assay, where the reduction of viable and metabolically-active cells is particularly evident and significant for the analysed nGD NPCs and for CBE-treated cells (Fig. 3b). These data confirm the hypothesis that the GCase enzyme deficit induces a decrease in cell growth due to a defective neural cell proliferation possibly coupled with increased cell death.

To assess cell death pathways, early and late apoptosis rates were measured through flow cytometry. After 9 days in culture, NPCs from GD lines showed an overall cell death rate higher than control lines, with a significantly increased percentage of PI-positive cells when compared with the matched control in the GD-2 line (Fig. 3c). Notably, the protein ratio between cleaved and total Caspase 3 was increased in GD-1, GD-3, and CBE-treated cells, thus confirming that these lines are more subjected to apoptotic stimuli (Fig. 3d).

### The Hippo pathway is activated in iPSC-derived neural GD cells

One of the main cell mechanisms involved in cell growth balance is the Hippo pathway, controlling cell proliferation, death, and differentiation. Considering that the hyper-activation of the Hippo kinase core leads to the downregulation of proliferating and antiapoptotic genes, its regulation can play a crucial role also in the neuronopathic GD condition.

**Fig. 4:**
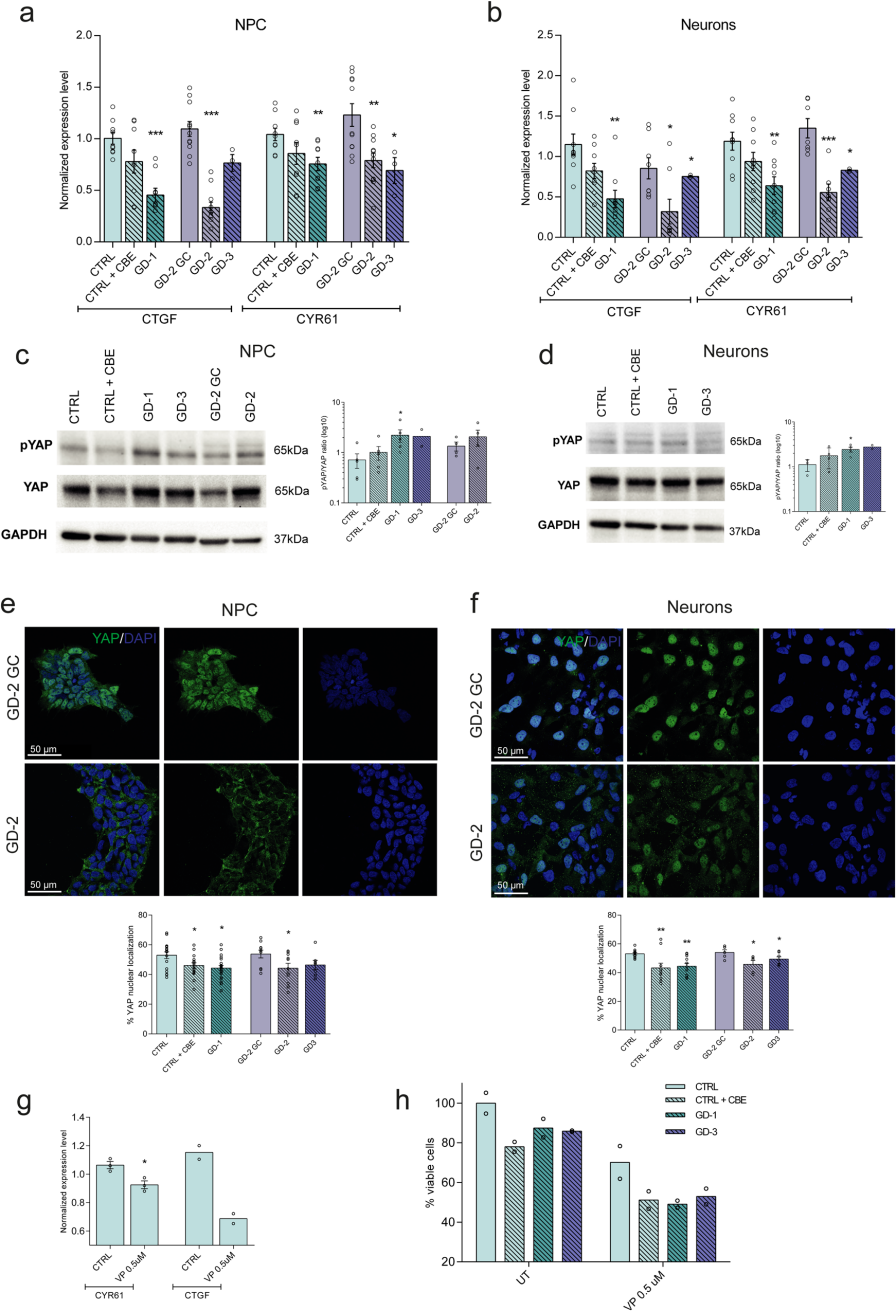
Hippo pathway is hyper-activated in GD NPCs and neurons. **a**, **b** Hippo target genes, CTGF and CYR61 mRNA levels in NPCs (**a**) and neurons (**b**) measured by qRT-PCR expressed as fold changes and normalized with respect to three different housekeeping genes (*n* = 3). **c** Representative Western blots of phosphorylated YAP (pYAP) and total YAP in all the NPC lines and quantification of pYAP/YAP ratio (*n* = 3). In the comparison CTRL vs GD-1 and vs the neuronopathic line GD-3, the increase is statistically significant. **d** Representative Western blots of phosphorylated YAP (pYAP) and total YAP in CTRL, CBE-treated, GD-1 and GD-3 neurons with the relative quantification (*n* = 3). pYAP/YAP ratio is significantly higher than the CTRL just for GD-3 neurons. **e**, **f** Representative images of YAP immunostaining (green) showing the cellular localization in the GD-2 and GD-2 GC iPSC-derived NPCs and neurons. Nuclei are counterstained with DAPI (blue). Scale bar: 50 µm. Quantification of the YAP nuclear localization in NPCs and neurons is shown for all the analysed lines (see Supplementary Fig. 5 for the other lines representative pictures) and was performed with the Intensity Ratio Nuclei Cytoplasm Tool plug-in of the Fiji software (*n* = 3). YAP nuclear localization is strongly reduced in GD-NPC and neurons. **g** qRT-PCR results of YAP transcriptional targets CTGF and CYR61 in CTRL NPCs after 24 h VP 0.5 µM treatment expressed as fold changes and normalized with respect to three housekeeping (*n* = 2). **h** MTT assay results, measuring cell proliferation and metabolic activity after 24 h of VP 0.5 µM treatment in NPCs (*n* = 2). All graphs show mean values and SEM. Statistical significance is indicated as *p* value (Student’s *t*-test), *p < 0.05, **p < 0.01, ***p < 0.001.

The expression level of the main human Hippo downstream targets, CTGF and CYR61, was analysed through qRT-PCR both in NPCs and neuronal cells. At the neural precursor stage, targets were significantly downregulated in the three GD lines (Fig. 4a), showing a similar effect also in terminally differentiated neurons (Fig. 4b). The same trend was observed for both CTGF and CYR61 also in the CTRL and CTRL + CBE comparison even if the difference is not significant. Certainly, the GD patient-derived lines are more reliable models to study GD pathway alterations, especially in isogenic conditions, while CBE treatment represents an approximation of the physiological situation. CBE causes indeed just a functional deficit of the GCase enzyme but is not able to reproduce the complex cellular environment due to *GBA* mutations. Therefore, these data suggest that the hyper-activation of the Hippo pathway core is particularly enhanced in the neuronopathic GD patient-derived cells. To confirm that the downregulation of these genes was strictly related to a reduction of the transcriptional activity of YAP, the pathway terminal effector, we evaluated its phosphorylation status and cytoplasmic retention, that is associated with the hyper-activation of the Hippo kinase cascade. The increased ratio between phosphorylated and total YAP is again especially evident in nGD cells (Fig. 4c, d), supporting the results obtained analysing the nuclear localization of YAP through immunofluorescence staining. A significant decrease in the nuclear localization of YAP emerged in GD-1 and GD-2 neural precursors (Fig. 4e and Supplementary Fig. 5a) and in all the analysed GD neurons (Fig. 4f and Supplementary Fig. 5b). No significant difference was detected at the NPC level between the GD-3 line and the GBA wild-type cells, whereas at neuron stage the decrease of nuclear YAP was significant in all patient lines, consistent with an enhancement of the hyper-Hippo condition, along with terminal differentiation during disease progression.

To test whether the modulation of the Hippo pathway in the GD context has an impact on the alteration of cell growth highlighted in our previous experiments, we treated CTRL, GD-1, and GD-3 NPCs with 0.5 µM Verteporfin (VP), the minimum concentration capable to induce a significant downregulation of YAP transcriptional activity in NPCs (Fig. 4g). VP is a second-generation photosensitizer initially approved for the treatment of age-related macular degeneration, that acts as an inhibitor of the interaction of YAP with its transcriptional partner TEAD, causing the block of the transcriptional activation of YAP downstream targets^55^. After 3 days in culture and 24 h treatment, VP-treated CTRL NPCs displayed a strong reduction of cell proliferation, comparable to the one observed in untreated GD NPCs. The VP effect on CBE-treated and GD NPCs caused an exacerbation of the growth proliferation, possibly caused by a further inhibition of YAP transcriptional activity with consequent cytoplasmic retention and degradation (Fig. 4h).

Taken together, these data support the view in which the Hippo pathway is hyper-activated in human iPSC-derived GD-neural progenitors and neurons and plays a potential role in nGD cell growth defects.

### Hyper-activation of Hippo signaling is confirmed in flies and Hpo-KD rescues the proliferative impairment

**Fig. 5:**
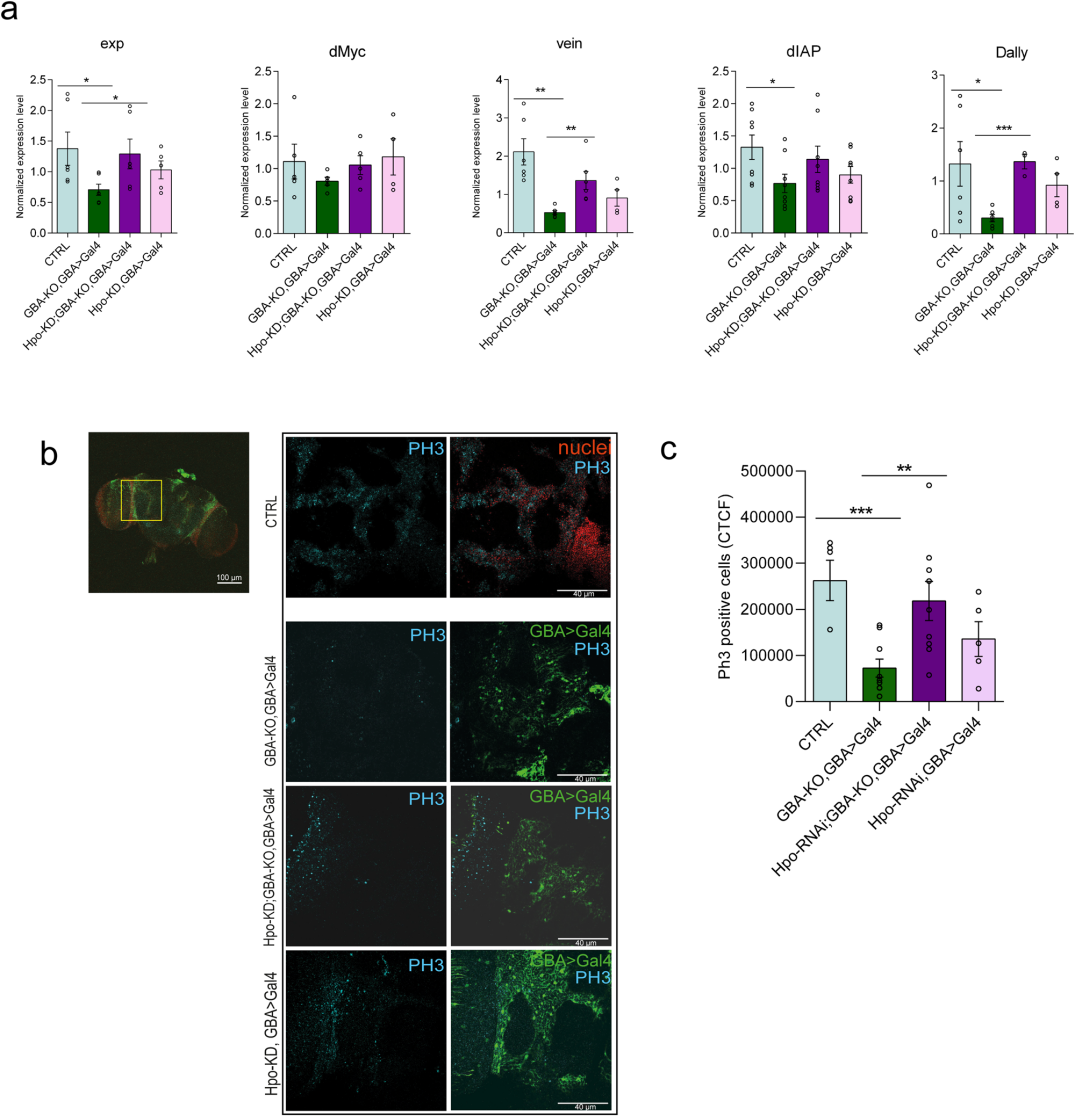
Hpo-KD rescues the proliferative impairment observed in GBA-KO adult brains. **a** In the GBA-KO heads the expression level of Yki targets expanded, dMyc, vein, dIAP, and dally, are decreased, whereas in the Hippo interference context, they are restored to wt levels. qRT-PCR performed on the RNA extracted from control flies (CTRL—w^1118^ in light blue), GBA-KO (in blue), Hpo interference GBA-KO (in purple), and Hpo-KD, GBA > Gal4 (in pink) heads three days after hatching. *p < 0.05; **p < 0.01; ***p < 0.001. **b** Immunofluorescence against PH3 performed on w^1118^ (CTRL), GBA-KO, GBA > Gal4; Hpo-KD, GBA-KO, GBA > Gal4, and Hpo-KD, GBA > Gal4. The analysed area of the fly brain is indicated with a square in a CTRL brain. Scale bar: 100 µm. The portion of the organ is zoomed in the images (60X magnification). PH3 staining is shown in cyan, while in the merged pictures, nuclei are in red for the CTRL and GBA > Gal4 cells are marked in green with the endogenous GFP. Scale bar: 40 µm. **c** Quantification of PH3 positive cells performed with the CTCF method. **p < 0.01, *** *p* < 0.001. All graphs show mean values and SEM. Statistical significance is indicated as *p* value (Student’s *t*-test).

To investigate the relationship between GBA loss and Hippo pathway activation in the neural tissue, we evaluated the expression in the fly brains of some of the main Yki targets such as expanded, dMyc, vein, dIAP, and dally^56–58^ (Fig. 5a). Expanded is commonly used as a readout of the activity of the pathway, while dMyc, vein, and dIAP are involved in cell proliferation and differentiation, and dally is a proteoglycan acting as a morphogen and deeply involved in cell-cell communication. The experiment was conducted in the GBA-KO flies and in the same flies in a Hpo-KD background. In order to verify whether the effect on Yki targets expression and cell proliferation are not related to the activation of the Hippo pathway only but refer also to the GBA-KO context, we performed the experiments also in Hpo-KD, GBA > Gal4 flies, in which the downregulation of the Hippo kinase is promoted in a GBA heterozygous model. GBA-KO flies show strong inhibition of Yki targets expression, supporting the hypothesis of a hyper-activation of the Hippo pathway. On the contrary, Hpo knockdown flies show a partial or total recovery in the expression of these genes as compared to the wt levels.

We then examined the proliferative rate in adult fly brains. Cell proliferation, as assessed by PH3 staining, is severely impaired in GBA-KO, GBA > Gal4, while Hpo-KD rescues cell proliferation in the GBA-KO, GBA > Gal4 background to the levels of control wild-type flies (Fig. 5b, c). Notably, in the Hpo-KD control flies, the recovery in the expression of the Yki targets and in the proliferative rate do not reach the same levels of the GBA-KO rescued flies.

To summarize, these data demonstrate that Hippo hyper-activation contributes to the growth defects observed in the GBA-LoF context, suggesting that targeting the Hippo pathway can be a promising therapeutic strategy in nGD.

## Discussion

Despite the great improvements achieved in the last decades in the treatment and management of GD patients, different aspects of the pathology still remain challenging. In particular, GD type 2 and 3 patients are orphans of effective therapies, and the only available interventions are focused on the management of symptoms.

Little is known about the molecular events leading to the different phenotypic manifestations and driving the wide clinical spectrum deriving from a common mutational event in the *GBA1* gene. Also, the lack of correlation between the residual GCase activity and disease severity^6^ supports the hypothesis that other modifier genes exist underlying the phenotypic variation.

A number of modulators of residual enzyme activity have been proposed as determinants of disease severity, including the lysosomal GCase activator Saposin C^59^, the lysosomal integral membrane protein type 2 (LIMP-2), identified as a sorting receptor for GCase^60^ and levels of ER retention and ER-associated degradation in the proteasomes^61^.

Genome-wide association studies have been performed to identify new modifiers in Ashkenazi Jewish patients with type 1 GD, homozygous for N370S mutation, and in GD mice models treated with CBE^62,63^. Even if several single nucleotide polymorphisms (SNPs) and many genes controlling neuronal excitability, endo-lysosomal function, and brain development were found correlated with disease severity, the question regarding the role of all these targets in nGD altered cell processes remains unanswered.

A more mechanistic approach focused on the identification of signaling pathway alterations modifying GD proinflammatory conditions and neuronal degeneration are expected to convey a better understanding of the neuronopathic disease pathophysiology. To this end, we devised an integrated approach taking advantage of two different and complementary systems: *Drosophila melanogaster* and human iPSCs differentiated towards the neural fate, both recapitulating key nGD traits.

Through whole transcriptome sequencing of the *Drosophila* brain, we were able to identify signatures of pathways differentially expressed in the GBA-KO condition, including genes typically involved in the lysosome and proteasome functionality and, more interestingly, in regulating cell proliferation and cell death.

Our study firstly assessed the impairment of these fundamental processes both in *Drosophila* tissues and in the iPSC model, as can be inferred by the reduced cell areas in fat body tissues and by the reduced proliferation rate both in the fly adult brains and in iPSC-derived neurons. The damage to the physiological proliferation mechanism appears more and more frequently associated with the neurodegenerative pattern. Specifically, the Hippo pathway gained a lot of attention in the last years for its many functions in orchestrating tissue growth in adults, modulating cell proliferation, differentiation, and migration in developing organs and the nervous system^64–66^. Deregulation of the Hippo pathway can lead to aberrant cell growth and cancer development when downregulated (hypo-Hippo) or, on the contrary, to neurodegeneration when hyper-activated (hyper-Hippo)^67,68^. An impairment of the pathways promoting cell growth at the neuronal level was proven in our GD models and the Hippo pathway has been proposed as a possible active player.

YAP/TAZ in humans and Yki in flies are the final effectors of the Hippo signaling cascade controlling transcription of many different genes that are commonly used as read-outs of pathway activity. Our data demonstrate that the expression levels of these transcriptional targets are lower in GD iPSC-derived NPC and neurons, as well as in *Drosophila* GD adult brains. The hypothesis of a GD-related hyper-Hippo condition was also supported by the increased pYAP/YAP protein ratio and the reduction of YAP nuclear localization in the cellular model. At the same time, the rescue experiment performed in adult fly brains offers an indication of the possible use of the Hippo cascade as a target in nGD. Indeed, even if it is reasonable that hippo KD may cause a proliferative boost also in wt cells, it is of great relevance the effect evidenced in GBA-LoF cells where the proliferative recovery can rescue the neurodegenerative phenotype. Altogether these results explain the NPC decreased proliferation and mature neuron degeneration and indicate the hyper-Hippo condition as a possible key contributor to nGD phenotype. Indeed, hyper-activation of the Hippo pathway has been already reported in other neurodegenerative disorders such as Huntington’s Disease, amyotrophic lateral sclerosis, retinal degeneration, and Alzheimer’s disease^30,34,69^.

The wide number of interactions of the Hippo pathway with many cellular mechanisms makes it a really promising target; it was indeed reported to be involved in the control of inflammation, ER stress, and cell death, all mechanisms altered in GD^43,70,71^. Moreover, it is known to directly interact with some specific pathways impaired in GD models, such as mTOR, Wnt/β-catenin, and cathepsins activity^15,72,73^. Therefore, understanding the pathological implications of Hippo in GD is particularly appealing also because many pharmacological modulators targeting Hippo are currently under evaluation for clinical applications. Recent evidence shed light on the fundamental role played by *GBA1* in the regulation of extracellular vesicle trafficking, which has been demonstrated to be essential in modulating the neurodegenerative phenotype^74,75^. As Hippo is deeply involved in endocytosis and exocytosis mechanisms, we can speculate that the interplay between *GBA1* and Hippo signaling can, at least in part, be related to the impairment of this important mechanism in the GD pathological context.

In conclusion, the identification of growth/death unbalance and the putative correlation of the hyper-Hippo condition to *GBA1* loss has been verified in both our model systems, thus proving the efficacy of the combined experimental approach based on Drosophila knockout models and human-derived iPSC lines. Furthermore, the discovery of Hippo hyper-activation as a key pathway linked to *GBA1* loss-of-function represents a valuable chance to fill the gap in the knowledge of modulating factors driving the different nGD manifestations and to explore new strategies to target this disease.

## Methods

### Fly stocks, fly rearing, and genetic manipulation

Fly stocks used in this study are w^1118^ (indicated as CTRL); Gba1b^KO^ (GBA-KO), the homozygous knockout (KO) obtained from the L. Partidge Lab^22^; Gba1b-Gal4 (Bloomington Drosophila Stock Center—BDSC n°78943), that have been used in combination with one copy of GBA-KO, in order to create a heteroallelic KO with the Gal4 promoter (GBA-KO, GBA > Gal4).

To induce GBA KD, the following strains were used: *Gba1b*^KD^ (GBA-RNAi, BDSC n°38970); *Minute*, *engrailed*-Gal4, UAS-GFP, produced in our laboratory (*M,en*); *GMR*-Gal4 (BDSC n°1104); UAS-mCD8::GFP (BDSC n°5137); UAS-*hpo*^KD^ (Vienna Drosophila Resource Centre—VDRC n°104169); UAS-*luc*^KD^ (luc-RNAi BDSC n°35788) used as a reference in the RNAi experiments.

Each fly stock used was grown on the same medium at 25 °C. Only the experiments involving the *Minute* and *engrailed* flies were performed at 29 °C.

### Adult retina fluorescence evaluation

Using *GMR* > Gal4 as specific retina promoter and UAS-mCD8::GFP, as GFP driver, the head of the adult flies of *GBA*-KO, UAS-*GBA*-RNAi (one copy each), and UAS-*luc*-RNAi as control, were cut at different time points, in order to evaluate the fluorescence associated with ommatidia degeneration. The fluorescent retinas were imaged using a Nikon Eclipse 90i microscope, taking advantage of the Z-stack acquisition tool present in the Nis-element Software, and the quantification used the Corrected Total Cell Fluorescence (CTCF) method.

### Wing hair count and evaluation of fat body cells area

Adult wings from *Minute, engrailed* > Gal4; UAS-GFP/UAS-*GBA*-RNAi (*M,en*; *GBA1b*-RNAi) (experiment population) and *Minute, engrailed* > Gal4; UAS-GFP/UAS-*luc*-RNAi (*M,en*; *luc*-RNAi) individuals were mounted in Fluoromount media and observed under the Nikon Eclipse 90i microscope. Eight images were taken for each genotype using the 60X objective, always focusing on the distal vein area. For each picture, the hairs included in two random 3 × 3 cm squares, were counted. For the fat body cells area, 12 images for CTRL and GBA-KO samples were taken; in each one, three cells were measured using ImageJ Software.

### Climbing assay and survival rate

CTRL, *GBA*-KO, and *GBA-*KO, *GBA* > Gal4 flies for the climbing assays were separated at birth into males and females and kept 25 per vial at 25 °C. The climbing test was performed on days 3, 7, 15, and 20 after birth. An equal number of female and male flies was placed inside a 50 ml transparent glass cylinder and, once acclimated, the cylinder was tapped down hard enough to knock all the flies down to the bottom; after 10 s, the number of flies able to reach three pre-established levels (below 5 cm—between 5–7.5 cm—above 10 cm) was counted. Ten seconds is the time commonly used for this type of analysis, considering that a wild-type fly is able to reach the top of the tube in 5 s. The rank is functional in evaluating fly climbing capability. The protocol was repeated ten times at 5-min intervals. About 150 flies were used for the climbing assay, coming from three different crosses representing three independent biological replicates. For the survival analysis we employed 200 flies observed for 100 days. Survival analysis was performed by Kaplan–Meyer curve estimator and significance was assessed by log-rank test.

### iPSC lines generation and characterization

Two of the iPSC lines employed in this study have been generated from the peripheral blood mononuclear cells (PBMCs) of a healthy donor (CTRL) and a GD type 1 patient (GD-1) carrying a compound heterozygous condition for the N370S and L444P mutations^76^. Briefly, after blood collection, mononuclear cells have been isolated through Ficoll (Lymphoprep) gradient centrifugation, and infected with Sendai vectors encoding for the reprogramming factors: Klf4, cMyc, Sox2, and Oct4 from the CytoTune™-iPS 2.0 Sendai Reprogramming Kit (Thermo Fisher) according to the manufacturer instructions. After 2 weeks in culture, the first iPSC colonies started to emerge. The pluripotency of the cells has been tested in live staining by TRA 1-60 antibody (Thermo Fisher), and through flow cytometry evaluation of the TRA 1-81 positivity (BD). Only the positive colonies have been harvested and transferred in a new vitronectin-coated plate for amplification and further characterization. To check if any chromosome alteration occurred during reprogramming, the cell karyotype was evaluated before and after the process through the G-banding technique. The pluripotency of the cells was tested through the expression level analysis of Oct4, Nanog, and Sox2 and the evaluation of the embryoid bodies (EB) formation; single iPSCs were seeded in ultra-low attachment plates (StemCell) in Essential 6 medium (Thermo Fisher). After 7 days in culture, they spontaneously generated small body structures, which have been kept in culture for another week. The presence of the three germinal layers has been evaluated through quantitative Real-time PCR (qRT-PCR) performed on the RNA extracted from the EB structure after 14 days in culture. Primers for endoderm-, mesoderm- and neuroectoderm-specific genes were used (Supplementary Table 1).

The second GD line (GD-2) was derived from fibroblasts of a GD type 3 patient presenting an L444P homozygous mutation, that was already characterized and corrected by gene editing^17^.

The third GD line (GD-3) was reprogrammed from a second GD type 3 patient with *GBA1* L444P homozygous mutation, according to the following protocol. Fibroblasts were isolated from a 2 mm skin biopsy and cultured in standard conditions (DMEM + 15% FBS + 2 mM glutamine + 0.1 M non-essential amino acids + 1% penicillin/streptomycin). On the day of transduction, cells were incubated with reprogramming vectors overnight following the manufacturer’s instructions (CytoTune™-iPS 2.0 Sendai Reprogramming Kit, Thermo Fisher Scientific). Three weeks after transduction, iPSC colonies were transferred to six-well plates previously coated with Cultrex (R&D).

For immunofluorescence characterization, cells grown on borosilicate glass coverslips were rinsed with 1X PBS, fixed with 4% paraformaldehyde for 10 min, and rinsed twice with 1X PBS. Cells were then incubated in a blocking solution (1X PBS + 0.1% Triton X-100 + 5% normal donkey serum) at room temperature for 1 h. Blocking solution was replaced with primary antibody solution (1X PBS + 0.1% Triton X-100 + 1% normal donkey serum + primary antibodies at proper dilution: OCT4, Cell Signaling, 1:100; SSEA4, Cell Signaling, 1:200; SOX2, Cell Signaling, 1:200; TRA 1-60, Cell Signaling, 1:500) and cells were incubated at 4 °C overnight. The following day, cells were rinsed three times with 1X PBS + 0.1% Triton X-100. After a final wash, cells were incubated in secondary antibody solution (1X PBS + 0.1% Triton X-100 + 1% normal donkey serum + secondary antibodies at proper dilution: Alexa Fluor 488 anti-mouse IgG, Thermo Fisher Scientific, 1:500; Alexa Fluor 647 anti-rabbit IgG, Thermo Fisher Scientific, 1:500) and DAPI (1:1000) for 1 h at room temperature. Finally, cells were washed three times with 1X PBS + 0.1% Triton X-100 and slides were mounted onto imaging slides (Thermo Fisher Scientific). Slides were stored at 4 °C until image acquisition with the confocal microscope.

Karyotype analysis was performed as follows: cells were incubated with colchicine for 3 h and then with 0.6% sodium citrate and 0.13% potassium chloride. Cells were then fixed with methanol/acetic acid and incubated with Quinacrine solution. Metaphases were acquired and analyzed with MetaSyste-Ikaros.

The study protocol was approved by the Ethical Committee of S.Orsola-Malpighi Hospital of Bologna (code84/2019/Sper/AOUBo). Written informed consent was obtained from all study participants or their legal guardians.

### iPSC culture maintenance

iPSCs colonies were cultivated in Essential 8 medium (Thermo Fisher) on vitronectin-coated (Thermo Fisher) B35 plates (Falcon)^77^. For standard passaging, cells were washed once with D-PBS, incubated for 2 min in a solution of D-PBS with 0.5 mM EDTA, detached in 1 mL of fresh medium and transferred in a new coated plate. For procedures requiring the single cell condition, after D-PBS washing, cells were incubated with 1 mL of Accutase (Thermo Fisher) for 5 min at 37 °C. Once detached, Accutase was diluted with DMEM-F12 medium (Gibco) and centrifuged for 5 min at 300×*g* in a 15 mL tube. Cells were then resuspended in the final medium supplemented with ROCK inhibitor Y27632 10 µM (Miltenyi) according to the different protocols. iPSCs were frozen in KnockOut Serum (Thermo Fisher) with 10% DMSO. Cells were periodically tested for Mycoplasma.

To mimic *GBA* loss, a healthy donor-derived iPSC line was treated with the GCase inhibitor CBE at 100 µM concentration for 3 to 9 days, once differentiated into neural precursors or neurons.

### Differentiation of iPSC into NPCs and mDA neurons

To generate mature mDA neurons, iPSCs were first differentiated towards the neural precursor cell (NPC) fate^78^. Briefly, colonies were detached 3–4 days after splitting, resuspended in iPSC medium without FGF2, and supplemented with the small molecules: SB-431542 (10 µM—Tocris), dorsomorphin (1 µM—Tocris), CHIR-99021 (3 µM—Axon Medchem), purmorphamine (PMA, 0.5 µM—Merck Millipore), and transferred to a new plate. After 2 days, they started to form EB structure in suspension and the basal medium was supplemented with N2 and B27 supplements (Thermo Fisher) and the previously cited small molecules. After two other days in culture, the medium was changed to NPC expansion medium containing N2-B27, 150 μM ascorbic acid (AA; Sigma-Aldrich), 3 μM CHIR-99021, and 0.5 μM PMA. At the 6th day of the differentiation protocol, the EBs were collected, triturated, and seeded in a Matrigel-coated (Corning) 12-well plate in NPC maintenance medium with ROCK inhibitor Y27632 10 µM (Miltenyi). At this stage, cells express neural progenitor markers and, once disaggregated, form homogeneous colonies of neuroepithelial cells. To obtain mature mDA neurons, 70% confluent NPC were seeded in a six-well plate in the NPC maintenance medium. After 2 days in culture, the central nervous system differentiation stimulus was induced by increasing the PMA concentration to 1 µM and by adding 100 ng/mL FGF8 (Peprotech) to the N2-B27 medium. After 8 days in this differentiation medium, a cytokine cocktail containing: 20 ng/mL BDNF (Peprotech), 20 ng/mL GDNF (Peprotech), 1 ng/mL TGFβ3 (Peprotech), 500 µM dbcAMP (Appliochem), and 200 µM AA was added to induce the maturation of neurons. After 2 weeks in maturation conditions, with medium change every 3 days, neurons are ready for the experiments. Mature cells have been fixed for immunostaining and collected for RNA and protein extraction.

### iPSC-derived NPC growth rate and cell death evaluation

To evaluate the growth rate, NPCs were seeded at fixed cell densities (1 × 10^5^/well in a 12-well plate) in a standard maintenance culture medium and cell number was evaluated after 3, 6, and 9 days in culture. For the MTT assay (Roche), 10,000 NPCs have been seeded in a 96-well plate, and after 3 days in culture with NPC-specific medium, cell proliferation has been evaluated according to the kit protocol. The measurement of the released absorbance has been detected through the Spark multiplate reader (Tecan). When cells have been treated with 0.5 µM Verteporfin (Selleckchem), NPCs have been treated for 24 h before detecting cell proliferation through MTT assay.

Cell death and apoptosis were analysed through flow cytometry (MACSQuant Analyzer, Mylteni); NPC were detached with Accutase, washed once with PBS, and stained with propidium iodide (PI) and AnnexinV (FITC) for 30 min in PBS 4% FBS before data acquisition. Flow cytometry data were analysed with the FlowJo software.

### RNA extraction and RT-PCR

RNA extraction from *Drosophila* tissues was performed using the TRI Reagent® (Sigma-Aldrich), after mechanical disintegration and homogenization of the tissues. To avoid genomic contamination, the RNA samples underwent DNase treatment. To obtain cDNA, 250 ug of DNase-free RNA was reverse transcribed using the iScript Sigma Bio-Rad kit. *RPL32* was used as a housekeeping gene^22^. The quantitative-PCR protocol employed Sybr Green GreenER qPCR SuperMix (Invitrogen) according to the manufacturer’s protocol. Primer sequences are listed in Supplementary Table 2.

For iPSC-derived NPC and mDA neurons, total RNA was extracted by the RNeasy spin column method (Qiagen). 250 ug of RNA were reverse transcribed to cDNA through the Transcriptor first strand cDNA synthesis kit (Roche Diagnostics) with oligo-dT primers (2.5 μM). Quantitative Real-Time PCR (qRT-PCR) was performed with Fast Start Sybr Green Master Mix. (Roche Diagnostics) on the LightCycler 480 instrument (Roche Diagnostics) using specific primers for the target genes. GAPDH, ATPS, and RLP0 were used as housekeeping (hkg) genes (Supplementary Table 3).

For both models, DDCt method was used to quantify gene expression levels normalized on the hkg genes.

### Library preparation and whole transcriptome sequencing

Total RNA was extracted from three different biological replicates of *Drosophila* CTRL and *GBA*-KO brains each, at 3 and 15 days from hatching with TRI Reagent® (Sigma-Aldrich). cDNA libraries were synthesized starting from 500 ng total RNA with TruSeq stranded mRNA Sample Prep Kit v2 (Illumina, San Diego, CA, USA) following the manufacturer’s instructions.

Whole transcriptome sequencing was performed on Nextseq500 sequencer (Illumina) at 80 bp read length in paired-end mode, yielding a total of 76 GB and an average of 79,5 million reads per sample. Read pairs were mapped on the *Drosophila melanogaster* genome reference dm6 using the alignment program hisat2 (http://daehwankimlab.github.io/hisat2/). Raw counts were computed with htseq-count (https://www.ncbi.nlm.nih.gov/pmc/articles/PMC4287950/) using the Drosophila dm6 annotation Drosophila_melanogaster.BDGP6.95 from ENSEMBL (http://ftp.ensembl.org/pub/release-95/gtf/drosophila_melanogaster/). Raw counts were then normalized as count per million (CPM) by the R-Bioconductor package edgeR (https://bioconductor.org/packages/release/bioc/html/edgeR.html). The same package was adopted to estimate the differentially expressed genes (DEG) between conditions and time points. Upregulated and downregulated biological pathways were evaluated with the web tool FlyEnrichr (https://maayanlab.cloud/FlyEnrichr/).

### GCase enzyme activity

For the measurement of the enzymatic activity of GCase, the fluorescent substrate 4-methylumbeliferyl-*N*-acetyl-β-glucosamine (Sigma M-3633) was used. The test was performed on protein lysates, obtained by sonication in PBS + Triton X-100 0,01%. About 1 mg protein was incubated with 100 μL of substrate and water or CBE as a negative control. After 1 h at 37 °C, the fluorescence level was measured using the Spark multiplate reader (Tecan). Enzyme activity was expressed as nmol/mg/h.

### Dissection and immunofluorescence staining

The dissection of each analysed *Drosophila* tissue was performed following published protocols^79^. Samples were fixed in Paraformaldehyde (PFA) 3.7% for 20 min and permeabilized by washing the samples with PBS Triton (PBST) 0.3% solution. The antibodies were used at the following concentrations: rabbit PH3 (1:100, Upstate Technology), Cas3 (1:100 Cell Signalling Technologies); DAPI (Sigma) was used at the concentration of 2 ug/ml. Samples were mounted in Flouromount. The images were captured using a Leica TCS SP2 confocal microscope, or Nikon A1R confocal laser-scanning microscope, acquired with the 20X–40X air objective, and/or the 60X mineral oil objective. The magnifications used the Nyquist theorem not to exceed the zoom and capture false signals. The Fluoview software was used for acquisition and the Fiji (ImageJ) software for analysis.

iPSC-derived NPC and neurons were seeded on cover-glass at the density of 100,000 cells/well, fixed in 4% paraformaldehyde for 15 min at room temperature (RT), rinsed twice with PBS, and permeabilized using 0.25% Triton X-100 in PBS for 10 min at RT and blocked with 10% normal goat serum in PBS + 0.1% Triton X-100 for 1 h. After washing in PBS, the cells were subjected to a block of unspecific binding of the antibodies using 1% bovine serum albumin (BSA) prepared in PBS solution for 30 min at RT and subsequently incubated with primary antibody at 4 °C overnight (on). Then, samples were rinsed and stained with the appropriate species of Alexa Flour Fluor 488/568/ 546 conjugate secondary antibody (1:250; Invitrogen by Thermo Fisher Scientific) for 1 h at 37 °C in the dark. After that, nuclei were counterstained with ProLong™ Gold antifade reagent with DAPI (Invitrogen by Thermo Fisher Scientific) and stored at 4 °C in the dark.

Anti NeuN, a neuron-specific nuclear protein, primary antibody (Chemicon, Millipore) was used 1:100, anti-Nestin (Santa Cruz) 1:200, anti-β3-Tubulin (mouse, BioLegend, \#MMS-435P) 1:1000, anti-Tyrosine Hydroxylase (TH, rabbit, Pel-Freeze, \#P40101-150) 1:500, and YAP (rabbit, Cell Signaling Technology, \#14074) 1:200. Pictures were acquired with the Leica TCS SP8 confocal microscope (Leica), analyzed with Fiji software and brightness/contrast was adjusted equally. Nuclear localization of YAP in NPC and neurons was evaluated in 2 to 5 pictures per condition for each biological replicate with the Intensity Ratio Nuclei Cytoplasm Tool plug-in of the Fiji software.

### Western blotting

Protein lysates were obtained from cell pellets through sonication in PBS + Triton X-100 0.01% (three cycles of 10 s each) and quantified with the Pierce BCA protein assay kit (Life Technologies). About 20 ug of protein were loaded on a 12% gel for Western Blot analysis. Blots were incubated with primary antibodies (GBA—Abcam (1:750), pYAP—Cell Signalling Technologies (1:750), YAP—Cell Signalling Technologies (1:750), cleaved Caspase 3—Cell Signalling Technologies (1:1000), Caspase 3—Cell Signalling Technologies (1:1000), GAPDH—Cell Signalling Technologies (1:2000), β actin—Cell Signalling Technologies (1:5000)) overnight at 4 °C on a shaker platform and were then probed with anti-mouse and anti-rabbit (Cell Signalling Technologies) secondary antibodies (all 1:10,000) for 1 h at room temperature. Visualization was done by using Amersham ECL Western Blotting Detection Reagent (GE Healthcare). Densitometry analyses on the immunoblots were performed by ImageLab software (Bio-Rad).

### Statistics and reproducibility

Statistical testing involved two-tailed Student’s *t*-test, multiple *t*-test, one-way ANOVA, and log-rank test as indicated in each related figure legend. Data were expressed as mean ± SEM. The significance level was set at *p* value <0.05. To determine reproducibility, analysis involving flies, iPSC-derived NPC and neurons have been performed on at least three different biological replicates, except for VP treatments on NPC, where analysis on two biological replicates have been carried out. qPCR samples were run in three technical replicates. Sample size and replicates are stated in the corresponding figure legends. Data have been analysed with Prism 6 (GraphPad Software, San Diego, CA, USA).

### Reporting summary

Further information on research design is available in the Nature Portfolio Reporting Summary linked to this article.

## Supplementary information

Peer Review File

Supplementary Information

Description of Additional Supplementary Files

Supplementary Data 1

Reporting Summary

## Data Availability

The datasets generated and/or analysed during the current study and presented in the main figures are available as Supplementary Data 1. Uncropped and unedited blot images have been included as Supplementary Figs. 6,  7. Accession code for Drosophila RNA seq data deposited on NCBI Sequence Read Archive (SRA): BioProject ID PRJNA945014.
