# Supplementary material for: Neuronopathic Gaucher disease models reveal defects in cell growth promoted by Hippo pathway activation
Source: Commun Biol. 2023 Apr 19;6:431. doi: 10.1038/s42003-023-04813-2 (PMC10115838; doi:10.1038/s42003-023-04813-2)
Supplement: Supplementary file 2 — Supplementary Information [file 42003_2023_4813_MOESM2_ESM.pdf]

SUPPLEMENTARY TABLES

Supplementary Table 1. **Embryoid bodies primer list**. Primers used to check the expression levels of the pluripotency and germinal layers markers in the iPSC-derived EB.

Gene	Primer F	Primer R
NANOG	5' - CCATCCTTGCAAATGTCTTCTG - 3'	5' - CTTTGGGACTGGTGGAAGAATC - 3'
OCT4	5' - GCAGCAGATCAGCCACATC - 3'	5' - CTTGATCGCTTGCCCTTCT - 3'
SOX 2	5’ - TGCGAGCGCTGCACAT - 3’	5’ - GCAGCGTGTACTTATCCTTCTTCA - 3’
AFP1	5' - CAAAATGCGTTTCTCGTTGCT - 3'	R: 5' - GCTGCCATTTTTCTGGTGATG - 3'
FOXA2	5' - CCACCTGAAGCCGGAACA - 3'	5' - TGCTCCGAGGACATGAGGTT - 3'
GATA4	5' - GCTATGCGTCTCCCGTCAG - 3'	5' - GTGACTGTCGGCCAAGACC - 3'
HAND1	5' - AACTCAAGAAGGCGGATGG - 3'	5' - AGGGCAGGAGGAAAACCTT - 3'
MIXL1	5' - GGTACCCCGACATCCACTTG - 3'	5' - TAGCCAAAGGTTGGAAGGATTTC - 3'
NEUROD1	5' - GAGCACGAGGCAGACAAGAAG - 3'	5' - CCCCCGTTCCTCAGTGAGT - 3'
PAX6	5' - CCGCCCTGGTTGGTATCC - 3'	5' - TTGGTATTCTCTCCCCCTCCTT - 3'
SOX1	5' - ATGAAGGAGCACCCGGATTA - 3'	5' - CTTCTTGAGCAGCGTCTTGGT - 3'

Supplementary Table 2. ***Drosophila* primer list**. Target genes and primer sequences used for qRT-PCR analysis of Drosophila brains.

Gene	Primer F	Primer R
Rpl32	5' - ATCGGTTACGGATCGAACAA- 3'	5' - GACAATCTCCTTGCGCTTCT - 3'
dGAPDH	5’ - CTCGACTCACGGTCGTTTCA - 3’	5’ - GGTGATCTTCTGGCCGTTCA - 3’
dGba1b	5' - GAACCAGAGCAATCCCTTCA- 3'	5' - TCATCGAGAGTCACGTCCAC- 3'
myc	5' - AGCAAATCAGCAGGGAGCTT- 3'	5' - GGTTGAGCACATCGAGGACA- 3'
dIAP	5' - TCTCGGCCCGTATAGACACA - 3'	5' - CTGAAGTCGAAACTTGACGGC- 3'
dally	5' - ATTTGCGGCGGAAACTG - 3'	5' - TGGCCATTGCTGTCGTA - 3'
vein	5’- CGACATCTACAGGGAGCGAC - 3’	5’- TCTTCGTGACCTCTGCGTTC -3’
exp	5’- TGTGCTCATAGATGGCGAGTA - 3’	5’- CGCGAAAGTGTAGCTCCAGG -3’

Supplementary Table 3. **List of qRT-PCR primers for iPSC-derived cells**. Sequence of the primers used to evaluate the expression levels of Hippo pathways effectors and the relative housekeeping genes.

Gene	Primer F	Primer R
CTGF	5' - CCGCCTGTGCATGGTCA - 3'	5' - GGTGCAGCCAGAAAGCTCA - 3'
CYR61	5' - TTTGTGAGGTGCGGCCTT - 3'	5' - CTTGGGCCGGTATTTCTTCA - 3'
ATPS	5' - GTCTTCACAGTTCATATGGGGA - 3'	5' - ATGGGTCCCACCATATAGAAGG - 3'
GAPDH	5' - AGGGGAGATTCAGTCTGG - 3'	5' - CGACCACTTTGTCAAGCT - 3'
RLP0	5' - CCTCATATCCGGGGGAATGTG - 3'	5' - GCAGCACTGGCACCTTATTG - 3'

Supplementary Table 4. Gene enrichment analysis results. Table representing the significantly upregulated pathways comparing the expression profile of WT and GBA KO flies (adj p-value<0.03).

Term	Overlap	P-value	Adj P-value	Z-score	Combined Score	Genes
Toll and Imd signaling pathway	23/65	2,65E-12	2,36E-10	-25,58	681,86	kay;Def;PGRP-SA;pirk;PGRP-SC2;dl;spirit;SPE;PGRP-SD;PGRP-LC;PGRP-LB;nec;Myd88;IM3;IM2;PGRP-LF;pll;grass;Rel;Jra;p38c;key;p38a
Endocytosis	31/121	7,90E-12	3,52E-10	-1,38	35,29	Khc;Hrs;Su(dx);Stam;Vps2;Hsc70-4;Snx1;cpb;cpa;Arpc3B;Arpc3A;Rho1;Snx6;Cdc42;CG10103;Vps60;Rab10;Arpc1;ALiX;Vta1;Vps20;Arpc2;Chmp1;InR;Usp8;Mvb12;Vps28;shrb;CHMP2B;Arpc5;Arpc4
Proteasome	15/52	3,71E-07	1,10E-05	-13,27	196,43	Rpn5;Pomp;Rpt6;Rpt6R;Rpt4R;Prosbeta6;Rpn8;Prosbeta7;Prosbeta4;Prosbeta3;Prosalpha3;Prosalpha4;Prosalpha5;Rpt4;Rpt5
AGE-RAGE signaling pathway in diabetic complications	9/31	7,78E-05	0,002	-69,21	654,80	RhoL;Col4a1;Rel;Jra;Rac2;Ras85D;p38c;Cdc42;p38a
Lysosome	19/118	0,0001	0,002	-0,96	8,63	Gba1a;Mvl;CG30160;Tsp42Ee;Cp1;Tsp42Ed;Lip4;Tsp42Eb;CG4250;Npc2h;LManI;CG42566;cathD;CG3376;Sap-r;CG12163;CG15117;Ppt1;CG18278
MAPK signaling pathway	16/93	0,0002	0,003	-2,02	17,42	kay;vap;Ask1;dos;raw;edl;Shc;chic;RhoL;Jra;Rac2;Ras85D;fs(1)M3;p38c;puc;p38a
Apoptosis	13/69	0,0003	0,004	-1,15	9,35	kay;Dronc;Ask1;Strica;Drice;LamC;Jra;Tspo;Dcp-1;Ras85D;fzo;Buffy;puc
Phagosome	15/89	0,0004	0,004	-8,09	64,12	Act5C;Act42A;Cp1;betaTub56D;mys;Act88F;Act87E;Sdic2;Hrs;VhaM9,7-b;betaTub60D;Itgbn;RhoL;Rac2;Act57B
ECM-receptor interaction	5/12	0,0005	0,005	-40,03	303,98	CG3168;Itgbn;LanB2;Col4a1;mys
Sphingolipid metabolism	7/28	0,0013	0,012	-28,89	191,73	CG11425;Gba1a;schlank;CG3376;CG11426;ifc;bwa
Circadian rhythm	4/9	0,0015	0,012	-45,04	294,27	Pdp1;tim;per;vri
FoxO signaling pathway	11/65	0,0021	0,014	-31,04	191,97	CG5059;CG11658;polo;Gadd45;CycB3;Ras85D;Ilp6;InR;p38c;Pdk1;p38a
Phototransduction	8/38	0,0020	0,014	-6,84	42,54	CG30054;CG17760;Act5C;Act42A;CG13526;Act88F;Act87E;Act57B
Homologous recombination	6/23	0,0023	0,015	-67,75	411,60	spn-D;Rad51D;Blm;RPA3;RpA-70;RPA2
Fanconi anemia pathway	6/25	0,0036	0,022	-32,22	181,10	spn-D;Blm;Fancd2;RPA3;RpA-70;RPA2
DNA replication	7/35	0,0051	0,027	-39,31	207,52	PCNA;dpa;RPA3;RpA-70;RPA2;DNApol-alpha50;DNApol-alpha180
Hippo signaling pathway	10/63	0,0052	0,027	-6,69	35,15	Act5C;Act42A;Act88F;Act87E;CycE;Pak3;upd3;Act57B;l(2)gl;upd2

SUPPLEMENTARY FIGURES

a

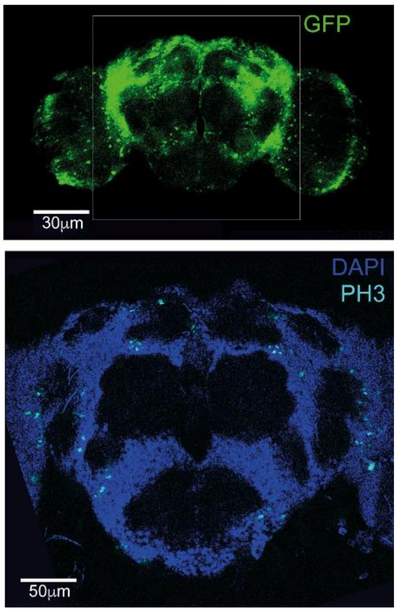

Supplementary Figure 1. a. Immunofluorescence on adult brains for PH3 performed on w^{1118} (CTRL) flies. The wild-type GBA expression pattern in GBA-Gal4, UAS-GFP adult brain is shown. The larger square highlights the central brain, the subregion analysed in the figures of the paper. PH3 is shown in cyan on CTRL brains. Nuclei are counterstained with DAPI. All the images represent single dorsomedial stacks of the adult brains.

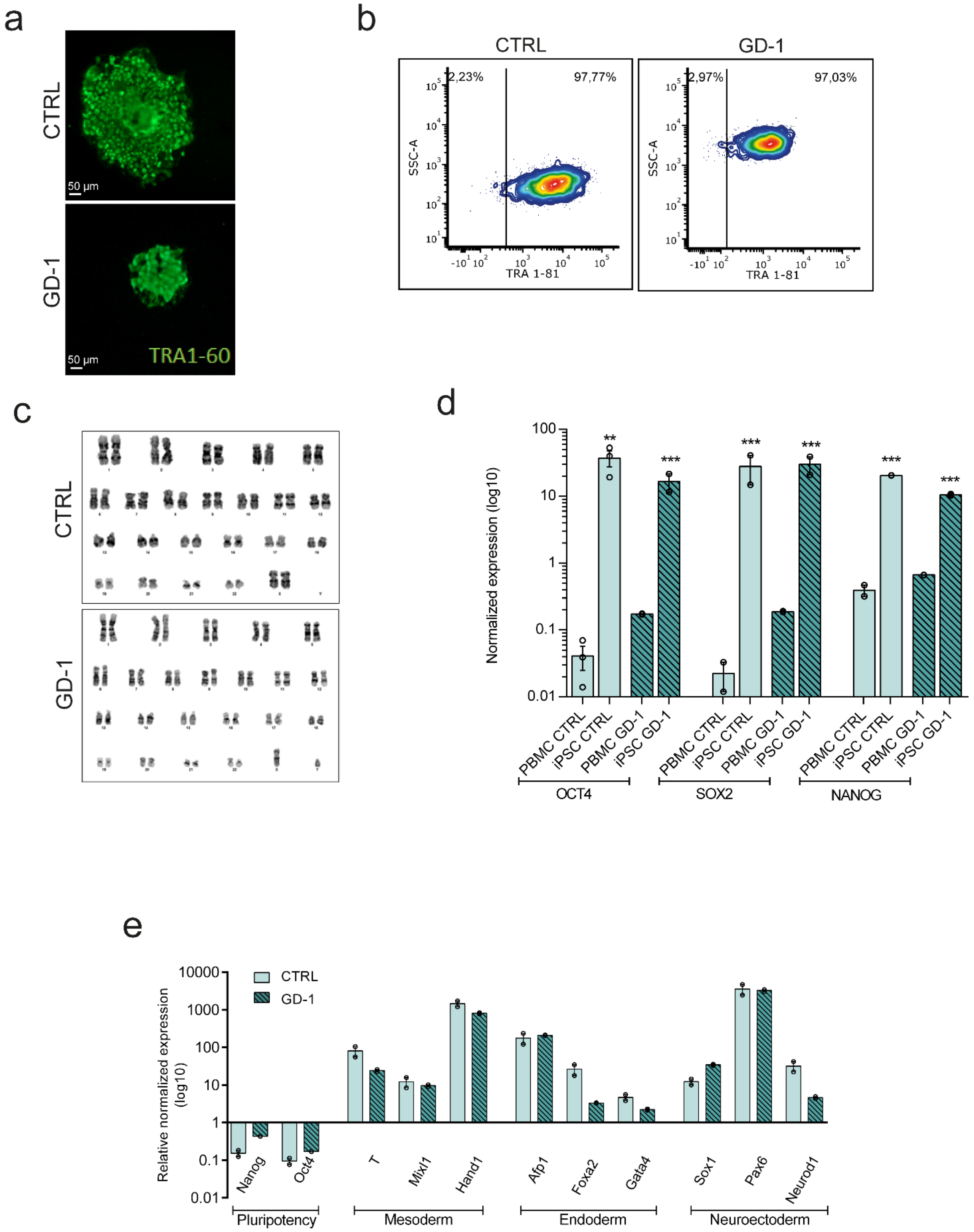

Supplementary Figure 2. a. Representative picture of an iPSC colony derived from the reprogramming of blood mononuclear cells of a healthy donor (CTRL) and a GD patient (GD-1). The anti-surface protein TRA1-

60 antibody was used in live staining to prove cells pluripotency. Scale bar: 50 µm. **b.** Dot plot representation of the flow cytometry analysis of TRA 1-81 positivity for CTRL and GD-1 iPSC. **c.** Karyotype of the two lines after the reprogramming process. **d.** mRNA levels measured through qRT-PCR of three pluripotency markers (Oct4, Sox2, Nanog) in CTRL and GD-1 cells. The expression levels after the iPSC reprogramming have been compared with the one in PBMC. Results are normalized with respect to three housekeeping genes (n=3). **e.** Expression levels of the three embryonic layer markers (mesoderm, endoderm and neuroectoderm) evaluated through qRT-PCR after spontaneous formation of embryoid bodies structures in both healthy donor and GD type 1 iPSC (n=2). While the pluripotency markers Nanog and Oct4 decrease in both populations, all the analysed germ lineage markers increased in the embryoid body state. All data are normalized on the expression level of the markers at iPSC state. Results are expressed as fold changes and normalized with respect to three housekeeping genes. Statistical significance is indicated as *p*-value (Student's t-test), ** $p<0.01$ and *** $p<0.001$. Graphs show mean values and SEM.

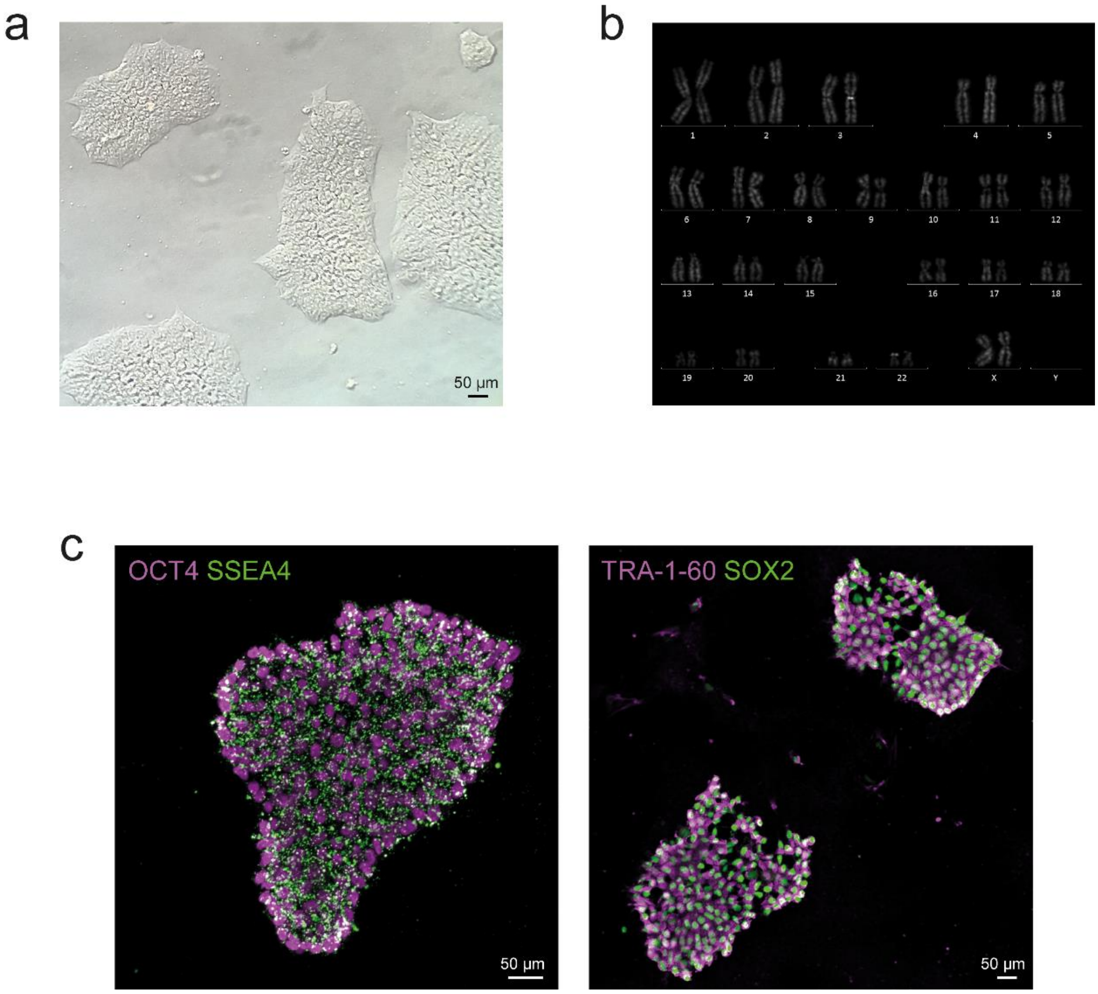

Supplementary Figure 3. a. Representative brightfield image of GD-3 iPSC colonies. **b.** Karyogram of the *GBA*-mutant iPSC line (GD-3) after the reprogramming process occurred. **c.** Representative immunocytochemistry images of iPSC colonies showing expression of pluripotent stem cell markers (OCT4, SSEA4, SOX2, TRA160). Scale bar: 50 µm.

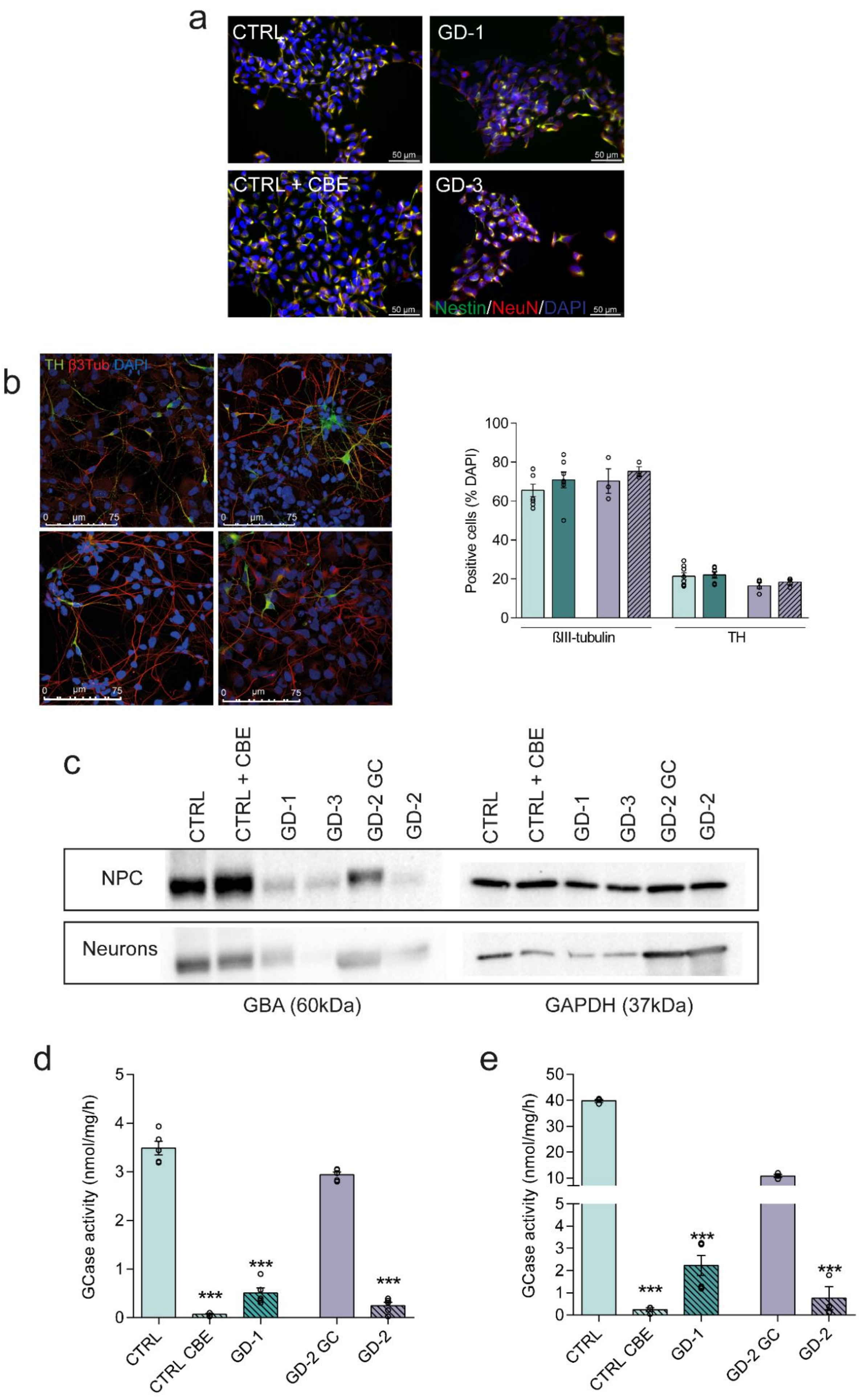
a
CTRL
GD-1
CTRL + CBE
GD-3
Nestin/NeuN/DAPI
50 µm
b
TH β3Tub DAPI
0 µm 75
Positive cells (% DAPI)
100
80
60
40
20
0
ßIII-tubulin
TH
c
CTRL
CTRL + CBE
GD-1
GD-3
GD-2 GC
GD-2
NPC
Neurons
GBA (60kDa)
GAPDH (37kDa)
d
GCase activity (nmol/mg/h)
5
4
3
2
1
0
***
CTRL
CTRL CBE
GD-1
GD-2 GC
GD-2
e
GCase activity (nmol/mg/h)
50
40
30
20
10
5
4
3
2
1
0
***
CTRL
CTRL CBE
GD-1
GD-2 GC
GD-2

Supplementary Figure 4. a. Immunostainings of the newly differentiated lines at neural precursor stage for Nestin (green) and NeuN (red). Nuclei are counterstained with DAPI (blue). Scale bar: 50 µm. **b.** Representative immunofluorescence pictures of terminally differentiated enriched mDA neurons (24 days of differentiation from the NPC stage). Cells are stained for β3tubulin (neuronal marker, red) and TH (dopaminergic neuron marker, green). Nuclei are counterstained with DAPI (blue). Scale bar: 75µm. Quantification of the positivity for the two markers shows no difference in differentiation efficiency among the four cell lines. **c.** Western blot evaluation of GCase protein levels in CTRL, CTRL treated with CBE, GD-1, GD-2, GD-2 GC and GD-3 at neural precursors and neurons levels**. d-e.** GCase activity measurement proving the strongly reduced enzyme activity in the CBE-treated and patient-derived lines (n=4). Statistical significance is indicated as *p*-value (Student's t-test), *** $p<0.001$. All graphs show mean values and SEM.

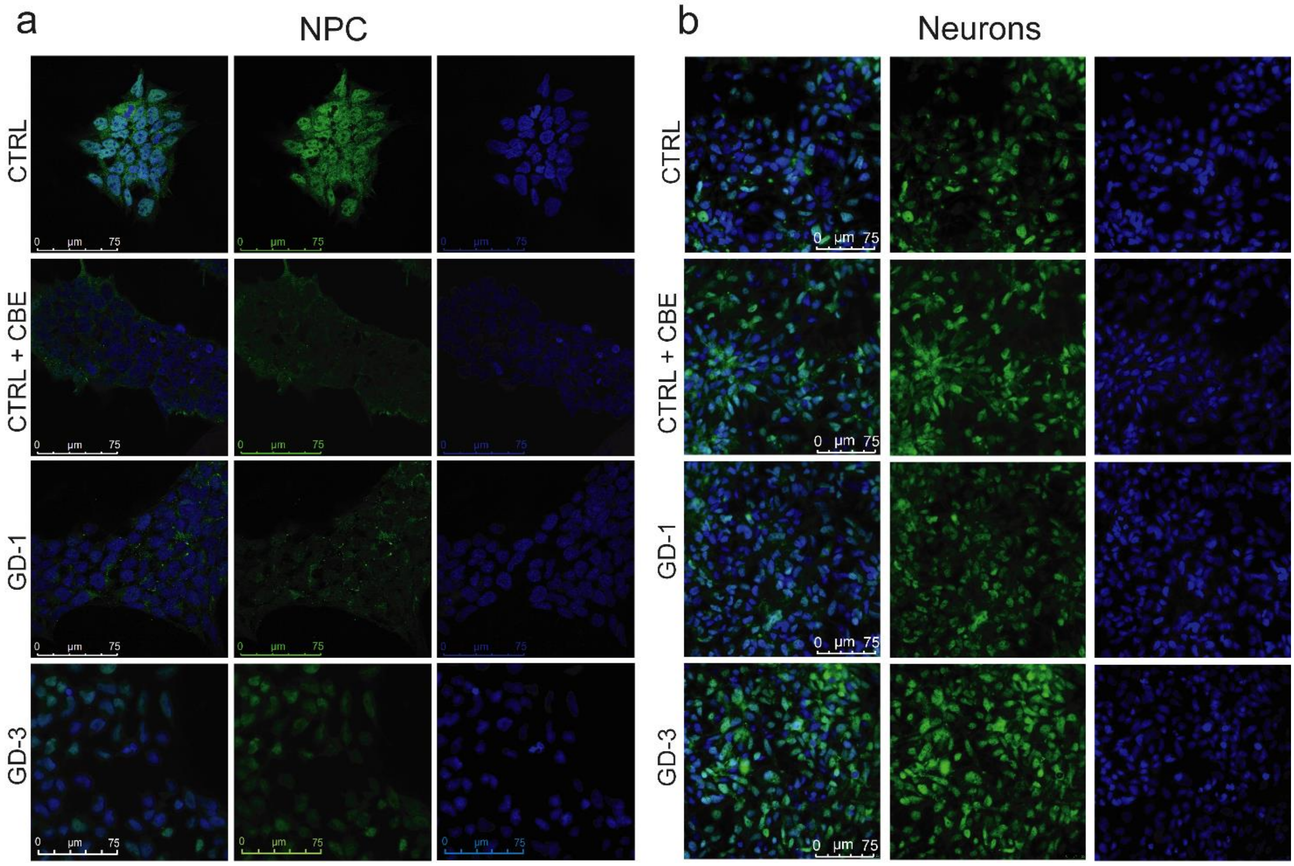

Supplementary Figure 5. a. Representative images of NPCs immunostained for YAP (green) and nuclear-stained for DAPI (blue). Scale bar: 75 µm. **b.** Representative images of neuronal cells immunostained for YAP (green) and nuclear-stained for DAPI (blue). Scale bar: 75 µm.

Blots in Figure 3d

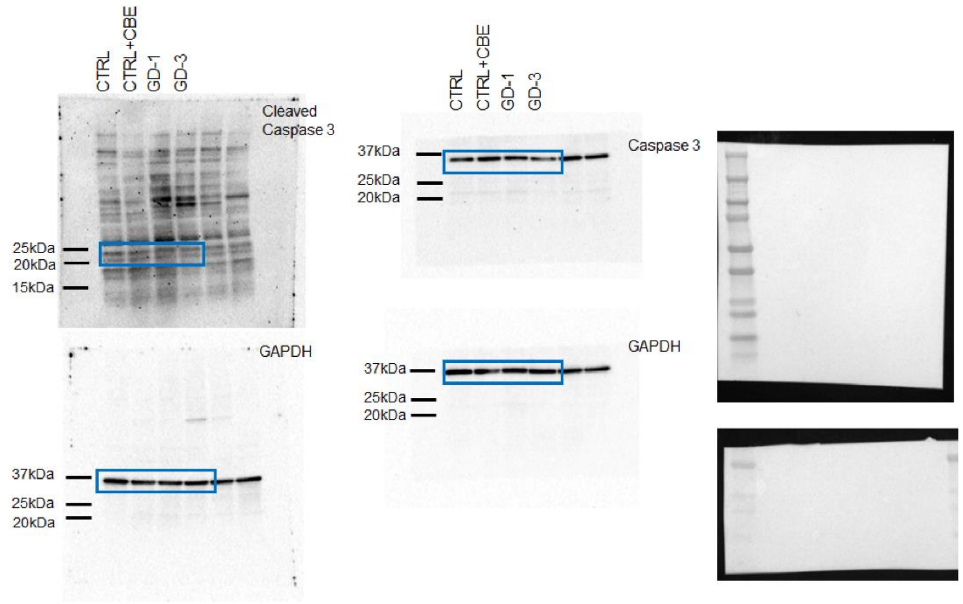

Blots in Figure 4c

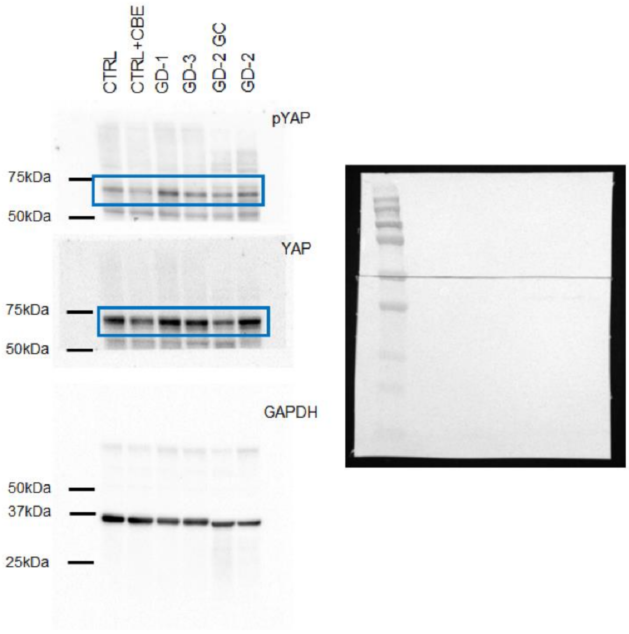

Blots in Figure 4d

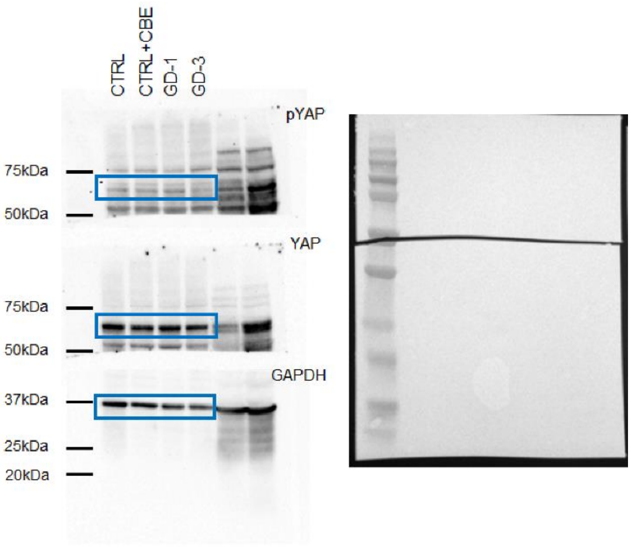

Supplementary Figure 6. Uncropped and unedited blot images present in the main figures of the manuscript.

Blots in Supplemetary Figure 4c

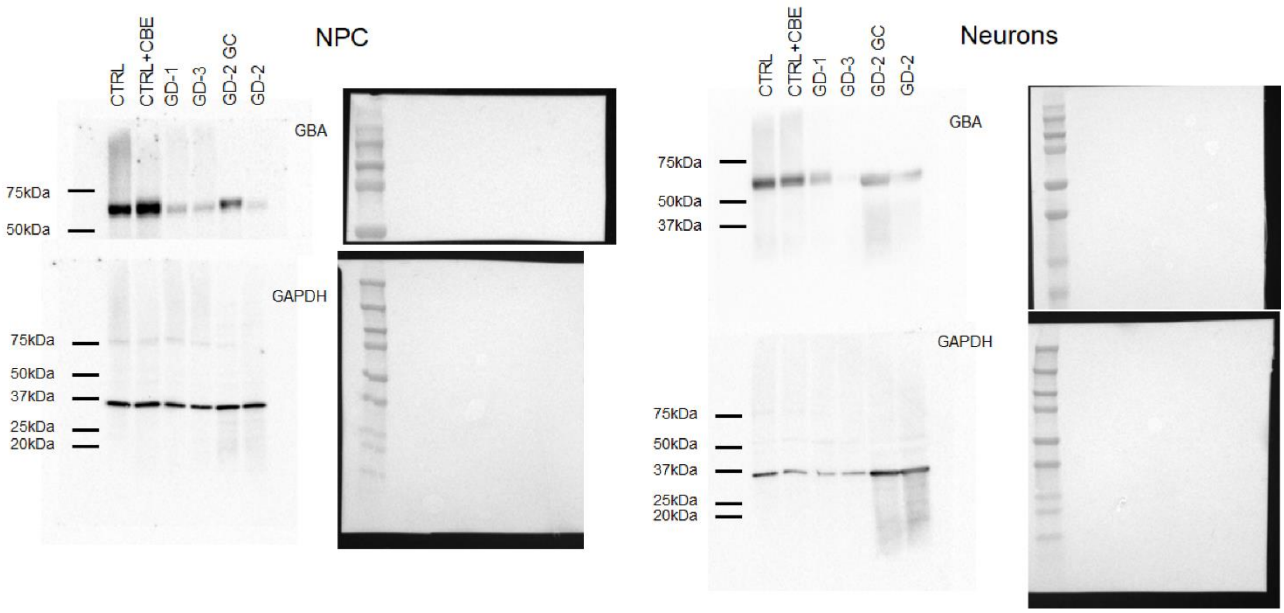

Supplementary Figure 7. Uncropped and unedited blot images present in the supplementary figures of the manuscript.
