# Supplementary material for: Neuronopathic Gaucher disease models reveal defects in cell growth promoted by Hippo pathway activation
Source: Commun Biol. 2023 Apr 19;6:431. doi: 10.1038/s42003-023-04813-2 (PMC10115838; doi:10.1038/s42003-023-04813-2)
Supplement: Supplementary file 3 — Description of Additional Supplementary Files [file 42003_2023_4813_MOESM3_ESM.pdf]

Description of Additional Supplementary Files

File Name: Supplementary Data 1

Description: The source data behind the graphs in the main figures of the paper.
