# Supplementary material for: Neuronopathic Gaucher disease models reveal defects in cell growth promoted by Hippo pathway activation
Source: Commun Biol. 2023 Apr 19;6:431. doi: 10.1038/s42003-023-04813-2 (PMC10115838; doi:10.1038/s42003-023-04813-2)
Supplement: Supplementary file 5 — Reporting Summary [file 42003_2023_4813_MOESM5_ESM.pdf]

nature portfolio

Corresponding author(s): Daniela Grifoni

Last updated by author(s): 04/04/2023

Reporting Summary

Nature Portfolio wishes to improve the reproducibility of the work that we publish. This form provides structure for consistency and transparency in reporting. For further information on Nature Portfolio policies, see our Editorial Policies and the Editorial Policy Checklist.

Statistics

For all statistical analyses, confirm that the following items are present in the figure legend, table legend, main text, or Methods section.

n/a	Confirmed	
☐	☒	The exact sample size (*n*) for each experimental group/condition, given as a discrete number and unit of measurement
☐	☒	A statement on whether measurements were taken from distinct samples or whether the same sample was measured repeatedly
☐	☒	The statistical test(s) used AND whether they are one- or two-sided<br>*Only common tests should be described solely by name; describe more complex techniques in the Methods section.*
☒	☐	A description of all covariates tested
☒	☐	A description of any assumptions or corrections, such as tests of normality and adjustment for multiple comparisons
☐	☒	A full description of the statistical parameters including central tendency (e.g. means) or other basic estimates (e.g. regression coefficient) AND variation (e.g. standard deviation) or associated estimates of uncertainty (e.g. confidence intervals)
☐	☒	For null hypothesis testing, the test statistic (e.g. *F*, *t*, *r*) with confidence intervals, effect sizes, degrees of freedom and *P* value noted<br>*Give P values as exact values whenever suitable.*
☒	☐	For Bayesian analysis, information on the choice of priors and Markov chain Monte Carlo settings
☒	☐	For hierarchical and complex designs, identification of the appropriate level for tests and full reporting of outcomes
☒	☐	Estimates of effect sizes (e.g. Cohen's *d*, Pearson's *r*), indicating how they were calculated

Our web collection on statistics for biologists contains articles on many of the points above.

Software and code

Policy information about availability of computer code

Data collection	Drosophila melanogaster genome reference dm6<br>dm6 annotation Drosophila_melanogaster.BDGP6.95
Data analysis	bcl2fastq - v2.20.0.422<br>hisat2 - v2.1.0<br>samtools - v1.7<br>htseq-count - v0.6.1p1<br>R - v3.6<br>R-bioconductor - v3.9<br>edgeR - v3.26.8<br>pheatmap - v1.0.12<br>FlyEnrichr - https://maayanlab.cloud/FlyEnrichr/<br>Prism 6 (GraphPad software)

For manuscripts utilizing custom algorithms or software that are central to the research but not yet described in published literature, software must be made available to editors and reviewers. We strongly encourage code deposition in a community repository (e.g. GitHub). See the Nature Portfolio guidelines for submitting code & software for further information.

Data

Policy information about availability of data

All manuscripts must include a data availability statement. This statement should provide the following information, where applicable:

- Accession codes, unique identifiers, or web links for publicly available datasets
- A description of any restrictions on data availability
- For clinical datasets or third party data, please ensure that the statement adheres to our policy

The datasets generated and/or analysed during the current study and presented in the main figures are available as Supplementary Data 1.
Accession code for Drosophila RNA seq data deposited on NCBI Sequence Read Archive (SRA): BioProject ID PRJNA945014.

Human research participants

Policy information about studies involving human research participants and Sex and Gender in Research.

Reporting on sex and gender	Sex and gender have not been considered in this study, including just three Gaucher disease patients-derived cells and an healthy donor. It is beyond the aim of this work a gender stratification of such a low number of people included.
Population characteristics	Patients affected with Gaucher disease, between 5 and 40 years old, carring GBA mutations (N370S/L444P and L444P/L444P) and an healthy donor.
Recruitment	Patients affected with Gaucher disease, that are between 5 and 40 years old, that have signed the informed consent. Given the disease rarity, only three patients affected with Gaucher disease with two different GBA mutation profile have been employed.
Ethics oversight	Ethical Committee of S.Orsola-Malpighi Hospital of Bologna (code84/2019/Sper/AOUBo).

Note that full information on the approval of the study protocol must also be provided in the manuscript.

Field-specific reporting

Please select the one below that is the best fit for your research. If you are not sure, read the appropriate sections before making your selection.

☒ Life sciences ☐ Behavioural & social sciences ☐ Ecological, evolutionary & environmental sciences

For a reference copy of the document with all sections, see nature.com/documents/nr-reporting-summary-flat.pdf

Life sciences study design

All studies must disclose on these points even when the disclosure is negative.

Sample size	Drosophila analysis: for behavioural assay >150 flies have been evaluated in order to minimize the variability of this type of tests. For the same reason the transcriptome sequencing was performed on the RNA extracted from > 50 flies for each replicate. In the other experiments at leat 10 individuals have been evaluated; notably, always female in order to do not incur in sex bias due to different genetic, phenotypic and beahavioural backgrouds.<br>iPSC lines have been generated from three patients and an healthy donor according to the patients material availability. Three different comparison between patient and the wild type counter part lines has been performed.
Data exclusions	In q-RT-PCR analyses some outlayers data have been removed when they were completely out of scale (for techical reasons).
Replication	All experiment have been performed at least three times, exept for VP treatments on neural precursors cells where the experiment have been performed two times.
Randomization	Randomization is not relevant since the work is based on comparison between case (pathological line or kd/ko model) and control (healthy or gene correctd counterpart/wild type flies) in different conditions.
Blinding	Blinding was performed for data analysis but not for data acquisistion since it was foundamental the distinction between case and controls.

Reporting for specific materials, systems and methods

We require information from authors about some types of materials, experimental systems and methods used in many studies. Here, indicate whether each material, system or method listed is relevant to your study. If you are not sure if a list item applies to your research, read the appropriate section before selecting a response.

Materials & experimental systems

n/a	Involved in the study
[ ]	[x] Antibodies
[x]	[ ] Eukaryotic cell lines
[x]	[ ] Palaeontology and archaeology
[ ]	[x] Animals and other organisms
[x]	[ ] Clinical data
[x]	[ ] Dual use research of concern

Methods

n/a	Involved in the study
[x]	[ ] ChIP-seq
[ ]	[x] Flow cytometry
[x]	[ ] MRI-based neuroimaging

Antibodies

Antibodies used	Drosophila experiments antibodies for immunofluorescent stainings: mouse βGal (1:500 DSHB), rat CycE (1:500 H. Richardson), rabbit PH3 (1:100, Upstate Technology), Cas3 (1:100 Cell Signalling Technologies).<br>iPSC-derived NPC and neurons antibodies for immunofluorescent stainings and WB: with DAPI (Biozol - 1:10000), β3-Tubulin (mouse, BioLegend, \#MMS-435P) 1:1000, Tyrosine Hydroxylase (TH, rabbit, Pel-Freeze, \#P40101-150) 1:500, YAP (rabbit, Cell Signaling Technology, \#14074) 1:100, GBA - Abcam, 1:1000, β actin - Cell Signalling Technologies, 1:5000, Anti NeuN (Chemicon, Millipore) 1:100, anti- Nestin (Santa Cruz) 1:200, anti β3-Tubulin (mouse, BioLegend, \#MMS-435P) 1:1000, cleaved Caspase3 - Cell Signalling Technologies #9664T (1:1000), Caspase 3 - Cell Signalling Technologies #9662 (1:1000), GAPDH - Cell Signalling Technologies #8884 (1:2000).
Validation	See the manufacturer website for each antibody.

Animals and other research organisms

Policy information about studies involving animals; ARRIVE guidelines recommended for reporting animal research, and Sex and Gender in Research

Laboratory animals	The study does not include live vertebrates and/or higher invertebrates.
Wild animals	The study does not include wild animals.
Reporting on sex	The study has been conducted on female flies. Female and male flies are characterized dy different behaviour and morphology that may result in different experimental outcomes. As the study is focused on the analysis of the molecular background of Gaucher disease, and the sindrome hit on female and male patient without a correlation with sex, we decided to select only female flies in order to avoid the bias associated with male specific gene expression and phenotype.
Field-collected samples	The study does not include samples collected from the field.
Ethics oversight	No ethical approval or guidance was required as the study took advantage of Drosophila melanogaster model, which falls into 3Rs principle.

Note that full information on the approval of the study protocol must also be provided in the manuscript.

Flow Cytometry

Plots

Confirm that:

- [x] The axis labels state the marker and fluorochrome used (e.g. CD4-FITC).
- [x] The axis scales are clearly visible. Include numbers along axes only for bottom left plot of group (a 'group' is an analysis of identical markers).
- [x] All plots are contour plots with outliers or pseudocolor plots.
- [x] A numerical value for number of cells or percentage (with statistics) is provided.

Methodology

Sample preparation	iPSC-derived human neural precursor cells detached with Accutase, washed once with PBS and stained with propidium iodide (PI) and AnnexinV (FITC) for 30 minutes in PBS 4% FBS.
Instrument	MACSQuant Analyzer, Myltenyi
Software	Flow cytometry data were analysed with the FlowJo software.
Cell population abundance	The whole cell population has been taken into account into the analysis.

Gating strategy

The analysis of Annexin/PI cells was peformed on structurally inteact cells, gated through physical parameters (FSC and SSC).

☐ Tick this box to confirm that a figure exemplifying the gating strategy is provided in the Supplementary Information.
