# Supplementary material for: Neuronopathic Gaucher disease models reveal defects in cell growth promoted by Hippo pathway activation
Source: Commun Biol. 2023 Apr 19;6:431. doi: 10.1038/s42003-023-04813-2 (PMC10115838; doi:10.1038/s42003-023-04813-2)
Supplement: Supplementary file 1 — Peer Review File [file 42003_2023_4813_MOESM1_ESM.pdf]

nature portfolio

Peer Review File

Open Access This file is licensed under a Creative Commons Attribution 4.0 International License, which permits use, sharing, adaptation, distribution and reproduction in any medium or format, as long as you give appropriate credit to the original author(s) and the source, provide a link to the Creative Commons license, and indicate if changes were made. In the cases where the authors are anonymous, such as is the case for the reports of anonymous peer reviewers, author attribution should be to 'Anonymous Referee' followed by a clear attribution to the source work. The images or other third party material in this file are included in the article's Creative Commons license, unless indicated otherwise in a credit line to the material. If material is not included in the article's Creative Commons license and your intended use is not permitted by statutory regulation or exceeds the permitted use, you will need to obtain permission directly from the copyright holder. To view a copy of this license, visit http://creativecommons.org/licenses/by/4.0/.

Reviewers' comments:

Reviewer #1 (Remarks to the Author):

This manuscript uses a combination of Drosophila genetics and IF, together with and human cell lines to present data in support of the conclusion that deregulation of the Hippo pathway contributes to the lysosomal Gaucher's disease (GD). There are well established links between vesicle and membrane traffic, which are altered in GD deficient cells, and the activity of the Hippo effector Yap1/Yki.

The data begin with a strong set of quantitative data in Figure 1 showing lifespan and neurodegenerative defects in whole GD null flies. Given the emphasis of the rest of the paper on GD-Hippo links, it is notable that none of these phenotypes are previously associated with Hippo. I understand and agree with the argument that GD loss does many other things, and that its loss should not be expected to fully phenocopy Yki reduced activity.

There are also no canonical Yki targets in the GMR eye disc RNAseq data in Fig 2. Where's the GD-Hippo link?

In addition, the pH3 and Caspase stains in Fig2 look grainy and are hard to conclude are true nuclei/cells without some sort of counterstain (dapi maybe?).

Figure 4 presents the strongest evidence of a Yap-GD link in human cells. the effect of GD-2 loss on Yap in NPCs is robust and suggestive of a mechanistic link from Yap to GD pathology

However, the data in Fig 5A-B which aim to carry this Yap-GD link into flies are insufficient to document/prove Yki activity is reduced. Steady state levels of proteins (e.g. CycE, Ex, dMyc, Diap1) are shown in fat body (FB) cells rather than any of the collection of Hippo transcriptional reporters used by the rest of the field e.g. ex-lacZ, diap-lacZ, ban-lacZ. Myc-lacZ. The Myc and dIAP data are not even shown in 5A – they must be to support the graph in 5B. And many other regulatory inputs (e.g. protein stability) likely affect the data in 5A. And why FB cells? The relevant defects are in wings and brain. Would that not be the place to link Yki/Hippo deregulation to cellular defects?

Second, the effect of CycE protein is very mild; it's not even clear why a protein normally expressed by cells as they transit G1 towards S would be expressed in post-mitotic FB cells.

Finally, the knockdown of Hpo rescuing reduced pH3 numbers in Fig5C-D in the brain could be very non-specific. Loss of Hpo drives most cells back into the cell cycle, so why would GD null cells be any different. The more significant question is whether control cells also have more pH3 after Hpo knockdown; if not, then the rescue gains credibility, but if so, then the effect does not imply that a reduced Yki drives the phenotype in GD cells.

As an aside, it could be very helpful to test whether reduced expression of Hpo, Warts, or Tgi rescue any of the organismal level defects observed in Fig1. That would provide broader relevance to the syndrome of GD phenotypes.

Reviewer #2 (Remarks to the Author):

In this manuscript by Strocchi et al., the authors investigated mechanisms contributing to neuropathic manifestations in Gaucher's disease using both Drosophila loss-of-function and human GD- iPSC models. Their results showed reduced proliferation and increased death of GD neuronal cells. The authors related this growth defect to deregulation of the Hippo pathway and downregulation of its transcriptional targets. The proliferative defect GBA-KO flies was rescued by Hippo knock-down

suggesting that hyper-activation of the Hippo kinase mediates the growth alterations in GD cells. The study is interesting and shed the light on the role of signaling pathways disrupted by GBA mutations in mediating neuropathic alterations in GD. However, there are several weaknesses in their use of GD iPSC model, which need to be addressed.

I have the following comments:
1- It is unclear why the authors choose to generate iPSCs from type 1 GD, if they are investigating the mechanisms leading to neuronopathic GD (Types 2 and 3). They also used another neuronopathic GD iPSC line but that's an (n) of one. I am not sure there are sufficient iPSC lines used in the study to make statistically significant claims of their mechanistic results.
2- The GD iPSC line they generated needs better characterization, the authors showed IF staining for one marker, TRA1-60. They need to show expression of the standard set of pluripotency markers such as SOX2, SSEA-3, SSEA-4, NANOG, TRA-1–81, and OCT4. They also need to test teratoma formation to prove pluripotency in vivo.
3- The authors mentioned that NPCs generated from EBs expressed neural progenitor markers, however they are not showing data or images reflective of NPCs characterization and the enrichment level of these markers.
4- In Figure s1D, the authors are showing TH and Tuj1 expression in GD dopaminergic neurons. According to their quantitation, although the majority of the cells are Tuj positive, TH is expressed by only ~20% of the cells, I don't think that is enough enrichment of Dopaminergic neurons to claim the results exclusively in this population. Also, TH expression alone is not sufficient for characterization of DA neurons, they need to characterize with other markers such as DAT, Foxa2,….
5- The authors need to characterize the growth defect in neuronal cells by cell cycle analysis as cell count alone is not an accurate indicator of cell proliferation status. Maybe it is the cell loss effect that gives these results and not the growth arrest.
6- The increased death of GD cells is interesting but needs to be better characterized by Western blot analysis for apoptotic markers.
7- In Figure 4 C, IF analysis for The nuclear localization of YAP in DA neurons is not convening as the images show almost similar colocalization of fluorescence signal in both GD2 and GD2 GC. Also if the quantitation for GD1 neurons are shown in the graph, you need to present corresponding images.
8- Since YAP phosphorylation status is critical in the proposed pathway, IF analysis is not sufficient to indicate the effect of GBA mutations, the authors need to use a phospho-specific antibody and show Western blots analysis and the corresponding quantitation.
9- To establish that that hyper-activation of the Hippo kinase is involved in neuronopathic complications in GD, the authors need to perform the Hippo knock-down experiment in iPSC neuronal cells and show the effect on growth and survival of these cells.
10- The figure legends need to be revised and include more information on data generation and analysis.

Response to Reviewers

Reviewer#1

This manuscript uses a combination of Drosophila genetics and IF, together with and human cell lines to present data in support of the conclusion that deregulation of the Hippo pathway contributes to the lysosomal Gaucher's disease (GD). There are well established links between vesicle and membrane traffic, which are altered in GD deficient cells, and the activity of the Hippo effector Yap1/Yki.
The data begin with a strong set of quantitative data in Figure 1 showing lifespan and neurodegenerative defects in whole GD null flies. Given the emphasis of the rest of the paper on GD-Hippo links, it is notable that none of these phenotypes are previously associated with Hippo. I understand and agree with the argument that GD loss does many other things, and that its loss should not be expected to fully phenocopy Yki reduced activity.

We thank the reviewer for this observation. We decided to focus on the behavioral and neuronal alterations in the context of GBA1b knock out to define the characteristics of our Drosophila model. Conversely, since it is well established that Hippo/Yki dosage can alter survival rates, we decided not to replicate the data (Huang J. et al., 2005). Hippo pathway deregulation in neurodegenerative mechanisms has been recently demonstrated in different contexts (Bruno et al., 2021). For example, in the Alzheimer disease (AD) lower expression of YAP resulted in an increase in Aβ1-42 and tau phosphorylation, as well as neuronal cell death in the initial stages of AD was also found to be a Hippo signaling pathway-dependent cell necrosis (Tanaka H. eta l., 2020; Xu M. et al., 2018). This point was better clarified in the Introduction section (pages 4-5, lines 84-94).

There are also no canonical Yki targets in the GMR eye disc RNAseq data in Fig 2. Where's the GD-Hippo link?

We agree with the reviewer that Figure 2 did not show explicitly the GD-Hippo link, but this was due to the fact that only the very top-scoring pathways were shown in the heatmap. Hippo signaling components were indeed among the significantly differentially expressed pathways and we extended table S4 to show the presence of Hippo signaling pathway among the over-represented ones. The reason why they are not among the top scoring could be related to the low expression of the hippo signaling pathway. Indeed, it has been demonstrated that hippo pathway components are deeply involved in both larval and adult neuronal brain cells proliferation and differentiation (Jukam D. et al., 2013; Jukam D. and Desplan C. 2011; Li G. et al., 2020; Vissers J. et al., 2015; Wittkorn E. et al.,2015), but the expression is limited to few cell populations.

In addition, the pH3 and Caspase stains in Fig2 look grainy and are hard to conclude are true nuclei/cells without some sort of counterstain (dapi maybe?).

Unfortunately, it was not possible to stain cells with dapi as the confocal Leica SP2 used does not include the UV lamp. To better show the colocalization of Ph3 within the nuclei we added a supplementary figure S5 as an example image of 2.5X magnification where the nuclei were stained with Propidium Iodide and the colocalization appeared as yellow dots.

Figure 4 presents the strongest evidence of a Yap-GD link in human cells. the effect of GD-2 loss on Yap in NPCs is robust and suggestive of a mechanistic link from Yap to GD pathology However, the data in Fig 5A-B which aim to carry this Yap-GD link into flies are insufficient to document/prove Yki activity is reduced. Steady state levels of proteins (e.g. CycE, Ex, dMyc, Diap1) are shown in fat body (FB) cells rather than any of the collection of Hippo transcriptional reporters used by the rest of the field e.g. ex-lacZ, diap-lacZ, ban-lacZ. Myc-lacZ. The Myc and dIAP data are not even shown in 5A – they must be to support the graph in 5B. And many other regulatory inputs (e.g. protein stability) likely affect the data in 5A. And why FB cells? The relevant defects are in wings and brain. Would that not be the place to link Yki/Hippo deregulation to cellular defects? Second, the effect of CycE protein is very mild; it's not even clear why a protein normally expressed by cells as they transit G1 towards S would be expressed in post-mitotic FB cells.

Thanks to the reviewer observations, we decided to remove the immunofluorescence on the Fat body cells, as this tissue may results out of the context of the proposed neurodegenerative model (page 10-11, lines 231-239). We therefore performed real time PCR analysis on multiple targets of the Hippo pathway, on adult brains in the wild-type context (w1118) and both in the GBA1b KO and Hippo interference context (figure 5A; page 31 line 628). Among the new targets we also included *Dally*, which is a target of the Hippo signaling pathway and a protein deeply involved in glial-to-neurons vesicles communications (Baena-Lopez L. A. et al., 2008) that could be suggestive of the possible altered mechanism in which hippo is involved.

Finally, the knockdown of Hpo rescuing reduced pH3 numbers in Fig5C-D in the brain could be very non-specific. Loss of Hpo drives most cells back into the cell cycle, so why would GD null cells be any different. The more significant question is whether control cells also have more pH3 after Hpo knockdown; if not, then the rescue gains credibility, but if so, then the effect does not imply that a reduced Yki drives the phenotype in GD cells.

The goal of this study was to identity the deregulated pathways in the neurodegenerative Gaucher in order to find new possible therapeutic targets. We found out that GBA1 loss in cells confers a growth defect that can lead to neurodegeneration. We then demonstrated the implication of Hippo signaling in this context and we hypothesized a connection with the proliferative deficit evidenced. We did not evaluate the effect of the hippo downregulation in the wt flies since our interest is focused on demonstrating that the hippo hyper-activation can at least in part justify the observed neurodegenerative phenotype. In these regards, hippo signaling may represent a new molecular target for this pathology. Notably our work is not the only one that shows how hippo knock down can recover a neurodegenerative phenotype: in the study of Gogia et al., the Authors identified hippo as a genetic modifier of FUS-mediated neurodegeneration, thus laying the basis of a novel molecular approach to fight Amyotrophic Lateral Sclerosis (Gogia N. et al., 2020). We added a comment on this point in the discussion section (page 13, lines 292-296).

As an aside, it could be very helpful to test whether reduced expression of Hpo, Warts, or Tgi rescue any of the organismal level defects observed in Fig1. That would provide broader relevance to the syndrome of GD phenotypes.

We agree that these are very good hints and suggestions, but unfortunately it resulted impossible to replicate the experiments of Figure 1 as they would have required long-time observation and actually in 2022 the Drosophila laboratory has been transferred to a new city and it is still not fully in operation.

Reviewer#2

In this manuscript by Strocchi et al., the authors investigated mechanisms contributing to neuropathic manifestations in Gaucher's disease using both Drosophila loss-of-function and human GD- iPSC models. Their results showed reduced proliferation and increased death of GD neuronal cells. The authors related this growth defect to deregulation of the Hippo pathway and downregulation of its transcriptional targets. The proliferative defect GBA-KO flies was rescued by Hippo knock-down suggesting that hyper-activation of the Hippo kinase mediates the growth alterations in GD cells. The study is interesting and shed the light on the role of signaling pathways disrupted by GBA mutations in mediating neuropathic alterations in GD. However, there are several weaknesses in their use of GD iPSC model, which need to be addressed. I have the following comments:

1- It is unclear why the authors choose to generate iPSCs from type 1 GD, if they are investigating the mechanisms leading to neuronopathic GD (Types 2 and 3). They also used another neuronopathic GD iPSC line but that's an (n) of one. I am not sure there are sufficient iPSC lines used in the study to make statistically significant claims of their mechanistic results.

The reviewer observation is highly reasonable. We decided indeed to introduce in the experimental plan a second neuronopathic GD iPSC line (GD-3). The line has been reprogrammed from a type 3 GD patient carrying the L444P homozygous mutation (page 8, lines: 167-168, page 17-18, 387-411). The data obtained on these cells are in line with the other neuronopathic GD line. We decided however to keep in our work also the GD type 1 line, considering that the behavior of this line seems to resemble in most of the experiment the neuronopathic lines one, even if with a smaller significance. This suggests that altered GCase functionality impacts on cells that do not show the GBA genetic status typical of nGD, thus confirming also at the cellular and molecular level the phenotypic continuum among the three GD isoforms.
Considering that we had not the possibility to repeat all the experiments involving the GD-2 GC and GD-2 line (just the ones requiring cell lysate and RNA), we did not always compare the new nGD line with the GD-2 line set but rather with the CTRL one.

2- The GD iPSC line they generated needs better characterization, the authors showed IF staining for one marker, TRA1-60. They need to show expression of the standard set of pluripotency markers such as SOX2, SSEA-3, SSEA-4, NANOG, TRA-1–81, and OCT4. They also need to test teratoma formation to prove pluripotency in vivo.

The lines characterization has been improved adding the quantification of Sox2, Nanog and Oct4 expression levels (Fig S1D) and the evaluation of TRA 1-81 surface markers expression by flow cytometry (Fig S1B), proving the actual pluripotent state of the employed cells. For ethical issues

we decided not to proceed with teratoma formation considering the results of *in vitro* characterization, especially the convincing outcomes of the embryoid body formation test.

3- The authors mentioned that NPCs generated from EBs expressed neural progenitor markers, however they are not showing data or images reflective of NPCs characterization and the enrichment level of these markers.

We used a well-established protocol for iPSC differentiation towards the neuronal fate (Reinhardt et al, 2013) that has been proven to give rise to NPC with a well characterized lineage specific expression profile. Nevertheless, we also added in Figure S3A the pictures of the immunofluorescence staining showing the expression of the NPCs markers Nestin and NeuN in all the newly differentiated lines (page 8, line: 169-170).

4- In Figure s1D, the authors are showing TH and Tuj1 expression in GD dopaminergic neurons. According to their quantitation, although the majority of the cells are Tuj positive, TH is expressed by only ~20% of the cells, I don't think that is enough enrichment of Dopaminergic neurons to claim the results exclusively in this population. Also, TH expression alone is not sufficient for characterization of DA neurons, they need to characterize with other markers such as DAT, Foxa2,….

The reviewer is right, the percentage of TH positive cells is rather low, even if also in the reference study the obtained positivity was around 30% (Reinhardt et al, 2013). We anyway decided to change the definition of our differentiated neuronal cells in enriched midbrain dopaminergic neurons (page 8, line: 171), not referring to them just as midbrain dopaminergic neurons.

5- The authors need to characterize the growth defect in neuronal cells by cell cycle analysis as cell count alone is not an accurate indicator of cell proliferation status. Maybe it is the cell loss effect that gives these results and not the growth arrest.

To better characterize the growth defect observed in neural cells, we carried out an MTT assay on four of our lines. This assay allowed us to measure cellular metabolic activity as a direct indicator of cell proliferation and viability. The obtained results (Figure 3B) confirmed the significant decrease in number of metabolically active cells in CBE treated and nGD patient derived-NPCs (page 8, lines: 176-180).
We also performed cell cycle analysis and checked through Western Blot the levels of phosphorylated and total cyclin D1, to evaluate a potential alteration in the cell cycle trends. This analysis showed no significant differences (data not shown).

6- The increased death of GD cells is interesting but needs to be better characterized by Western blot analysis for apoptotic markers.

Western Blot on cleaved-caspase3 and total caspase3 have been performed to evaluate the apoptosis activation in patients-derived and Gaucher-like NPC lines (Figure 3D). According to the results, CBE treated and GD lines have an increased cleaved/total Caspase 3 ratio, suggesting that

the induction of apoptosis is actually increased in GD NPC (page 8, lines: 185-187). Even if significant in 2 out of 3 conditions, we are aware that this effect is not exhaustive, suggesting that the increased apoptosis rate is probably not the only factor driving the growth defect seen in our GD lines.

7- In Figure 4 C, IF analysis for the nuclear localization of YAP in DA neurons is not convening as the images show almost similar colocalization of fluorescence signal in both GD2 and GD2 GC. Also if the quantitation for GD1 neurons are shown in the graph, you need to present corresponding images.

The representative pictures for neurons have been replaced with more informative ones. Certainly, the effect in not as evident as in NPCs, where the YAP signal intensity is stronger probably because of the higher proliferation status. It is indeed known that the expression of Hippo pathway components is particularly relevant at neural progenitor's cell level and in glia cells, more than in mature neurons (Ouyang et al, 2019). Representative pictures for the CTRL, CTRL+CBE and GD-1 conditions have been added in Figure S4A-B, as correctly pointed out by the reviewer, together with GD-3 line YAP staining and relative quantifications both for NPCs and neurons (page 9, lines: 208-213).

8- Since YAP phosphorylation status is critical in the proposed pathway, IF analysis is not sufficient to indicate the effect of GBA mutations, the authors need to use a phospho-specific antibody and show Western blots analysis and the corresponding quantitation.

YAP and pYAP Western Blot analysis have been performed and the results have now been added to the manuscript (page 9, lines: 206-208, Figure 4C-D). The results are in line with YAP localization analysis showing an increase of YAP phosphorylation in the analysed nGD and CBE treated NPCs and neurons. The increased pYAP/YAP ratio is an indicator of the reduction of YAP transcriptional activity that, once phosphorylated is not able to enter the nucleus and is retained into the cytoplasm or degraded.

9- To establish that that hyper-activation of the Hippo kinase is involved in neuronopathic complications in GD, the authors need to perform the Hippo knock-down experiment in iPSC neuronal cells and show the effect on growth and survival of these cells.

Since we had lab-related issues in performing lentiviral-mediated knock-down experiments, we decided to proceed with pharmacological modulation of the hippo pathway. There are indeed many tested molecules proved to interact with YAP and modulate its activity. The most widely used in literature is definitely verteporfin (VP), a small molecule acting as inhibitor of YAP/TEAD association and consequent YAP activity. We treated our NPCs with VP 0.5 µM and analysed the effect in terms of cell proliferation through MTT showing that VP-mediated YAP inhibition is able to provide an effect similar to GBA mutation in iPSC lines and to exacerbate the GD-line proliferation defect (page 10, lines: 214-224, Figure: 4G).

10- The figure legends need to be revised and include more information on data generation and analysis.

The figures legends have been implemented with more accurate descriptions, as requested by the reviewer.

Bibliography

Baena-Lopez LA, et al. *The tumor suppressor genes dachsous and fat modulate different signalling pathways by regulating dally and dally-like*. Proc Natl Acad Sci U S A. 2008 **105**(28):9645-50.

Bruno L, et al. *Mechanosensing and the Hippo Pathway in Microglia: A Potential Link to Alzheimer's Disease Pathogenesis?* Cells. 2021 **10**(11):3144.

Gogia N, et al. *Inactivation of Hippo and cJun-N-terminal Kinase (JNK) signaling mitigate FUS mediated neurodegeneration in vivo*. Neurobiol Dis. 2020 **140**:104837.

Huang J, et al. *The Hippo signaling pathway coordinately regulates cell proliferation and apoptosis by inactivating Yorkie, the Drosophila Homolog of YAP*. Cell. 2005 **122**(3):421-34.

Jukam D and Desplan C. *Binary regulation of Hippo pathway by Merlin/NF2, Kibra, Lgl, and Melted specifies and maintains postmitotic neuronal fate*. Dev Cell. 2011 **21**(5):874-87.

Jukam D, et al. *Opposite feedbacks in the Hippo pathway for growth control and neural fate*. Science. 2013 **342**(6155):1238016.

Li G and Hidalgo A. *Adult Neurogenesis in the Drosophila Brain: The Evidence and the Void*. Int J Mol Sci. 2020 **21**(18):6653.

Li G, et al. *A Toll-receptor map underlies structural brain plasticity*. Elife. 2020 **9**:e52743.

Tanaka, H., et al., *YAP-dependent necrosis occurs in early stages of Alzheimer's disease and regulates mouse model pathology*. Nat Commun. 2020 **11**(1):507.

Ouyang T., et al. *Recent Advances of the Hippo/YAP Signaling Pathway in Brain Development and Glioma.* Cell Mol Neurobiol. 2020 **40**(4):495-510.

Reinhardt P., et al. *Derivation and expansion using only small molecules of human neural progenitors for neurodegenerative disease modeling.* PLoS One. 2013. **8**(3): p. e59252.

Vissers JHA, et al. *The Scalloped and Nerfin-1 Transcription Factors Cooperate to Maintain Neuronal Cell Fate.* Cell Rep. 2018 **25**(6):1561-1576.e7

Wittkorn E, et al. *The Hippo pathway effector Yki downregulates Wg signaling to promote retinal differentiation in the Drosophila eye*. Development. 2015 **142**(11):2002-13.

Xu M, et al. *A systematic integrated analysis of brain expression profiles reveals YAP1 and other prioritized hub genes as important upstream regulators in Alzheimer's disease*. Alzheimers Dement. 2018 **14**(2):215-229.

Reviewers' comments:

Reviewer #1 (Remarks to the Author):

The authors have done a pretty good job in responding to my comments for clarification or additional experiments. Two elements of the data still need to be improved, and neither seem all that difficult to do.

The first one relates to the need for a counterstain in figures 2D-E to actually see where the cells are in the image. I suggested DAPI, which the authors explain is difficult to do. There are many other options; e.g., even a phalloidin/F-actin stain would suffice.

The second is to add a needed control genotype in Fig. 5 to show the effect of hippo-KD by itself on the qPCRs and pH3 stain. This serves as a positive control for the hippo-KD condition, and provides context for the "rescued" genetic combination.

Comment on the rebuttal to Reviewer #2's comments:

Thanks for the opportunity to reread the paper and view of all of the review comments. The authors seem to have added alot of data that was requested by reviewer #2. Many of Rev#2's requests were not based on doubts about the overall conclusions of the Gauchers-Hippo link, but rather for better explanations and needed controls or better images. There are some spots where more experiments would be helpful (e.g. Ga1 vs Ga2/3) but only to expand the data, not to rebut a concern. I remain somewhat disappointed that the authors responded to some Reviewer requests by explaining that they could not go back and reacquire new data, but Covid led to loss of time and reagents for labs around the world.

Overall, I am comfortable that its core findings are largely correct, and I am in favor of accepting the manuscript in its revised form.

Reviewer #1

The authors have done a pretty good job in responding to my comments for clarification or additional experiments. Two elements of the data still need to be improved, and neither seem all that difficult to do.

The first one relates to the need for a counterstain in figures 2D-E to actually see where the cells are in the image. I suggested DAPI, which the authors explain is difficult to do. There are many other options; e.g., even a phalloidin/F-actin stain would suffice.

We followed Reviewer's suggestion and repeated the IF analysis of the adult brains using a different confocal and stained for DAPI. We think this improves image quality and clarity (Figure 2D-E). We have also added in Figure S1 the representation of the GBA expression pattern in the whole *Drosophila* brain, as a further informative image to better understand why we focused the attention on the central brain, and a representative picture of a whole brain stained with PH3 and DAPI. The main text was modified accordingly to those changes (lines 153-160).

The second is to add a needed control genotype in Fig. 5 to show the effect of hippo-KD by itself on the qPCRs and pH3 stain. This serves as a positive control for the hippo-KD condition, and provides context for the "rescued" genetic combination.

We thank the reviewer for this suggestion. We added a Hippo-KD control in a GBA heterozygous context in our qRT-PCR and PH3 staining analysis reported in Figure 5. We decided to use UAS-Hpo KD with a copy of GBA>Gal4 as a driver to verify that the effect of Hippo interference on our GD KO model is tightly dependent on the KO context. Moreover, in this way we used the same Gal4 driver as in the experiment with GBA KO background avoiding possible effects related to the use of different Gal4 drivers. The Hpo-KD,GBA>Gal4 flies display and intermediate and non-significant effect in terms of Yorkie target expression level and PH3 protein expression, proving that the Hpo related rescue effect is strongly and specifically related to the lack of GBA (lines 234-247 and Figure 5).

Comment on the rebuttal to Reviewer #2's comments

Thanks for the opportunity to reread the paper and view of all of the review comments. The authors seem to have added a lot of data that was requested by reviewer #2. Many of Rev#2's requests were not based on doubts about the overall conclusions of the Gauchers-Hippo link, but rather for better explanations and needed controls or better images. There are some spots where more experiments would be helpful (e.g. Ga1 vs Ga2/3) but only to expand the data, not to rebut a concern. I remain somewhat disappointed that the authors responded to some Reviewer requests by explaining that they could not go back and reacquire new data, but Covid led to loss of time and reagents for labs around the world.

Overall, I am comfortable that its core findings are largely correct, and I am in favor of accepting the manuscript in its revised form.

We thank the reviewer for the positive comments and recommendation.

REVIEWERS' COMMENTS:

Reviewer #1 (Remarks to the Author):

The authors have responded to my request and provided additional data. I am in support of accepting & publishing the work.
